# Blood-activating and collateral-unblocking traditional Chinese medicine formulas combined with mecobalamin for peripheral neuropathy in type 2 diabetes: a systematic review and Bayesian network meta-analysis

**DOI:** 10.3389/fendo.2026.1888494

**Published:** 2026-09-16

**Authors:** Zijian Liu, Yue Wang, An Hu, Shaowen Jia, Tingyu Wu, Yuchen Fu, Hao Zheng, Luying Sun

**Affiliations:** 1 Department of Nephrology and Endocrinology, Dongzhimen Hospital, Beijing University of Chinese Medicine, Beijing, China; 2 School of Nursing, Beijing University of Chinese Medicine, Beijing, China; 3 Department of Nephrology, Fangshan Hospital, Beijing University of Chinese Medicine, Beijing, China

**Keywords:** blood-activating and collateral-unblocking therapy, diabetic peripheral neuropathy, mecobalamin, nerve conduction velocity, network meta-analysis, traditional Chinese medicine

## Abstract

**Background:**

Diabetic peripheral neuropathy (DPN) is a leading cause of neuropathic pain, disability and lower-limb amputation, and the pharmacological options currently available do little to reverse established nerve injury. Compound traditional Chinese medicine preparations that activate blood circulation and unblock the collaterals have shown promise as add-on therapy to neurotrophic treatment, improving nerve conduction and relieving symptoms, but head-to-head comparisons between individual formulas are lacking.

**Methods:**

PubMed, Embase, the Cochrane Library, CNKI, Wanfang and other databases were searched to July 2026 for randomised controlled trials comparing a blood-activating, collateral-unblocking formula plus mecobalamin with mecobalamin alone in adults with type 2 diabetes and peripheral neuropathy. The primary outcome was the Toronto Clinical Scoring System (TCSS); secondary outcomes were motor and sensory conduction velocity (MCV, SCV) of the median and common peroneal nerves, together with adverse events. Mean differences (MD) and 95% credible intervals were estimated in a Bayesian random-effects model, and ranking distributions were summarised as the surface under the cumulative ranking curve (SUCRA). Risk of bias was assessed with RoB 2 and certainty of evidence with CINeMA (Confidence in Network Meta-Analysis).

**Results:**

Nineteen randomised controlled trials (2,090 participants) were included. All were conducted in China, and most nodes were informed by a single trial. Relative to mecobalamin alone, most add-on regimens improved TCSS and nerve conduction velocity. Credible intervals excluded zero for 9 of 12 formulas for TCSS, 7 of 14 for median nerve MCV, 3 of 14 for median nerve SCV, 5 of 14 for peroneal nerve MCV and 8 of 9 for peroneal nerve SCV. By SUCRA, Yiqi Huashi Tongbi Formula ranked first for TCSS (90.3%; MD -3.49, 95% CrI -5.17 to -1.81), Shengyang Sanhuo Decoction for median nerve MCV (85.6%; MD 4.16, 95% CrI 1.58 to 6.78), Xiaoma Zhitong Capsule for median nerve SCV (84.4%; MD 4.19, 95% CrI 0.88 to 7.58), Modified Buyang Huanwu Decoction for peroneal nerve MCV (79.2%; MD 3.78, 95% CrI 0.83 to 6.72) and Jiangtang Tongluo Tablet for peroneal nerve SCV (95.2%; MD 4.08, 95% CrI 2.38 to 5.78). Eight of the 19 trials made no mention of adverse-event monitoring, and none reported numerical laboratory safety data. The available information is insufficient to evaluate the safety of these add-on regimens.

**Conclusion:**

Adding blood-activating, collateral-unblocking traditional Chinese medicine to mecobalamin was associated with improvement in TCSS and in nerve conduction velocity in patients with DPN. Certainty of evidence was very low for every comparison, however, and prediction intervals crossed the line of no effect for three outcomes, indicating that the direction of effect could change in a new study. The rankings are exploratory and hypothesis-generating: they are not a sufficient basis for inferring definite clinical efficacy or for choosing a formula for an individual patient, and they require confirmation in adequately powered, prospectively registered, blinded multicentre head-to-head trials.

**Systematic review registration:**

https://www.crd.york.ac.uk/PROSPERO/, identifier CRD42025123604.

## 1 Introduction

Diabetic peripheral neuropathy (DPN) is a length-dependent, symmetrical sensorimotor polyneuropathy driven jointly by chronic hyperglycaemia and cardiovascular risk factors (1). Its core pathology involves hyperglycaemia-induced metabolic disturbance, oxidative stress and inflammation (2), which together give rise to microvascular injury, nerve ischaemia and hypoxia, axonal degeneration, demyelination and downregulation of neurotrophic factors (2). Recent evidence also implicates exosomes in the pathogenesis of DPN, acting through neuroinflammation, Schwann cell function, axonal injury, intercellular communication and regenerative repair pathways (3).

Approximately 537 million adults worldwide have diabetes, and about half of them develop DPN (2, 4). Among Chinese patients with T2DM the prevalence of DPN reaches 67.6%, and the risk of disability rises with disease duration, particularly in older patients, in those with long-standing diabetes and in those with poor glycaemic control (5). Left untreated, DPN progresses irreversibly from subclinical abnormalities of nerve conduction to sensory loss, chronic pain and motor impairment, and ultimately to foot ulceration, infection and amputation (5, 6). Quality of life, psychological wellbeing and the capacity for independent living are often severely compromised (5, 6).

The pathogenesis of DPN spans several metabolic pathways (6): hyperglycaemia triggers activation of the polyol pathway, formation of advanced glycation end products, oxidative stress, inflammation and microvascular injury (6). Neural and vascular damage reinforce one another. Hyperglycaemia produces endothelial dysfunction, basement membrane thickening and microcirculatory obstruction, which reduce nerve blood flow and precipitate Schwann cell apoptosis together with axonal ischaemia, hypoxia and degeneration; the inflammatory mediators released after nerve injury in turn aggravate vascular inflammation and vasoconstriction (7). Protein glycation and oxidative stress driven by hyperglycaemia also disturb haemorheology, reducing erythrocyte membrane elasticity, increasing platelet reactivity and raising plasma viscosity (8), all of which compound local ischaemia. If sensory neuropathy is not controlled in time, motor and autonomic fibres become involved, manifesting as distal symmetrical polyneuropathy, gastrointestinal dysmotility and cardiovascular autonomic dysfunction; advanced disease may be complicated by foot ulceration, infection and Charcot arthropathy (9).

Comprehensive management of DPN has several components: tight control of glucose, blood pressure and lipids; symptomatic analgesia with agents such as pregabalin, gabapentin and amitriptyline; promotion of neural repair and functional maintenance with mecobalamin and vitamin B6; improvement of the microcirculation with alprostadil analogues and cilostazol; and antioxidant treatment with oral or intravenous alpha-lipoic acid (10). Of these, pregabalin, gabapentin and duloxetine are first-line agents for pain management in international guidelines, whereas alprostadil analogues, cilostazol and alpha-lipoic acid are used mainly in a subset of countries (10). Mecobalamin is an inexpensive and readily available active derivative of vitamin B12; pharmacological studies suggest that it may support the synthesis of myelin and axonal proteins, although direct evidence of structural neural repair in patients with DPN is still lacking. The drug nevertheless remains in wide use in Chinese clinical practice, particularly in primary care (11).

Each of these options has clear limitations. Because the pathological pathways of DPN are closely interwoven, conventional drugs directed at a single mechanism cannot provide comprehensive coverage: neurotrophic agents generally fail to reverse established structural nerve damage, and the evidence for their effect on nerve conduction velocity remains uncertain (12). Pharmacological analgesia is largely symptomatic; first-line agents such as duloxetine and pregabalin offer moderate efficacy but carry appreciable adverse effects, including somnolence, dizziness and gastrointestinal upset, while opioids are constrained by concerns about dependence and adverse events and are unsuitable for long-term management (10).

Although several validated screening and assessment tools for DPN are available, monofilament and vibration testing among them, their use in routine care is limited, and variable sensitivity, cost and accessibility further impede early, systematic and widespread identification of risk. Patients consequently tend to receive symptomatic treatment only once the disease has advanced and become irreversible (2, 9).

Against this background, blood-activating and collateral-unblocking traditional Chinese medicine has attracted growing interest as a component of comprehensive DPN management (13). Activating blood and unblocking the collaterals is a therapeutic approach defined within traditional Chinese medicine theory rather than a biomedical category. In this framework DPN is held to arise from blood stasis and obstruction of the vessels and collaterals, and treatment therefore commonly applies blood-activating, collateral-unblocking methods (14). Several studies suggest that such formulas, added to background therapy, may help improve nerve conduction and relieve the symptoms and signs of DPN (13).

Evidence on blood-activating, collateral-unblocking traditional Chinese medicine for DPN remains fragmented. Existing studies are predominantly pairwise designs comparing a single formula with conventional treatment, and direct evidence between formulas is absent. Because the trials differ in population characteristics, traditional Chinese medicine pattern classification and background treatment, these pairwise results cannot readily be set side by side. The present study therefore applies network meta-analysis to make indirect comparisons among multiple blood-activating, collateral-unblocking formulas anchored on a common comparator, and to rank them on an exploratory basis. It should be emphasised that, given the heterogeneity present and the star-shaped configuration of the current evidence network, in which most nodes consist of small single-centre trials, the rankings should be regarded on the present evidence as exploratory hypotheses rather than as definitive evidence.

## 2 Materials and methods

### 2.1 Study design and registration

This study is a systematic review and Bayesian NMA of RCTs, undertaken to compare the additional benefit conferred by blood-activating, collateral-unblocking traditional Chinese medicine formulas, given as add-on therapy to standard neurotrophic treatment, on clinical symptom burden and neurophysiological function in adults with T2DM and DPN. Reporting follows the PRISMA 2020 statement (15). The completed PRISMA 2020 checklist is provided in **Supplementary Table 1**. Eligibility criteria were specified in advance within the Population-Intervention-Comparison-Outcome-Study design (PICOS) framework, and the evidence base was restricted strictly to RCTs. The protocol was prospectively registered with PROSPERO (CRD42025123604) and the review was conducted accordingly. Registration was dated 20 November 2025 and was completed before screening and data extraction began. Amendments to the registered protocol were as follows: (1) the search was updated from 31 October 2025 to 31 July 2026; and (2) the fixed-effect model was replaced by a random-effects model. Eligibility criteria, outcomes and ranking methods were unchanged from the registered protocol.

### 2.2 Participants

The target population comprised adults with confirmed T2DM and DPN, with no restriction on sex, age or disease duration. Diagnosis required both a background of diabetes and evidence of peripheral nerve injury, established clinically or by nerve conduction testing, together with at least one of the following recognised standards: (1) the 1999 WHO diagnostic criteria for diabetes combined with clinical confirmation of peripheral neuropathy; (2) the American Diabetes Association (ADA) consensus on the diagnosis of DPN; (3) the Guidelines for the Prevention and Treatment of Type 2 Diabetes in China, issued by the Chinese Diabetes Society; or (4) the DPN diagnostic criteria set out in the Guiding Principles for Clinical Research on New Chinese Medicines. Only patients with T2DM were included, and type 1 diabetes was explicitly excluded in order to improve population homogeneity. To evaluate transitivity, we extracted the important effect modifiers across treatment nodes (age, sex, duration of diabetes, duration of DPN, baseline TCSS, baseline MCV and SCV, glycated haemoglobin, treatment duration, diagnostic criteria and co-interventions) and compared their distributions between nodes (**Supplementary Tables 3a–d**). Exclusion of peripheral neuropathy of other aetiologies was inconsistent across trials: exclusion of alcoholic neuropathy was reported in 5 trials, of compressive neuropathy in 3 and of neuropathy attributable to neurotoxic drugs in 3; renal insufficiency was explicitly excluded in 5 trials, while a further 8 referred only to general terms such as severe hepatic or renal dysfunction. None of the 19 trials reported screening for vitamin B12 deficiency or hypothyroidism.

### 2.3 Comparator and interventions

Control groups received oral mecobalamin as the background comparator. As reported in the primary studies, both arms received supportive background treatment, typically comprising dietary guidance, exercise advice and integrated control of glucose, blood pressure and lipids. Because the specific co-interventions differed between studies, however, background treatment should not be regarded as a fully standardised common co-intervention. Most primary studies also did not state explicitly whether the doses of these background agents, namely glucose-, blood pressure- and lipid-lowering drugs, remained stable during follow-up.

Intervention groups received a blood-activating, collateral-unblocking traditional Chinese medicine formula in addition to the control regimen, for 4 to 24 weeks. To keep the naming of interventions consistent and recognisable to an international readership, the Latin binomials of all constituent medicinal materials were standardised against the Pharmacopoeia of the People’s Republic of China (2020 Edition) (16) and the World Health Organization guidance on International Nonproprietary Names (INN) (17). Some formulas contain materials that require careful processing or raise toxicological concerns, including Aconitum species, Xixin (Asarum heterotropoides), Shuizhi (Whitmania pigra), Quanxie (Buthus martensii), Wugong (Scolopendra subspinipes mutilans), Tianlong (Gekko japonicus) and Dilong (Pheretima aspergillum); the individual species and doses are listed in **Table 1a**. None of the 19 trials, however, reported quality control information such as batch numbers of decoction pieces, content determination or chromatographic fingerprinting, and only 1 of the 5 in-hospital preparations provided both a preparation approval number and the crude drug content per unit. The name, full composition, dosage form and administration schedule of each formula are given in **Tables 1a**, **b**. Following the Pharmacopoeia of the People’s Republic of China (2020 Edition), a medicine was classified as a blood-activating, collateral-unblocking formula if either of two conditions was met: (1) the formula is explicitly defined as blood-activating and collateral-unblocking under its actions and indications; or (2) the formula contains materials whose stated actions include activating blood, resolving stasis (including dispelling, dissipating, expelling or breaking stasis) or unblocking the collaterals (including unblocking the channels), among them but not limited to Danshen (Salvia miltiorrhiza), Chuanxiong (Ligusticum chuanxiong), Honghua (Carthamus tinctorius), Chishao (Paeonia lactiflora), Dilong (Pheretima aspergillum), Shuizhi (Whitmania pigra) and Tubiechong (Eupolyphaga sinensis).

**Table 1a:** Composition of the included formulas.

Study	Formula	Node abbreviation	Required TCM pattern	Composition and dose
Shen 2009 (21)	Tangmaining Capsule	TMN	Blood stasis; qi deficiency	Shichangpu (*Acorus tatarinowii* Schott, Rhizoma), Gegen (*Pueraria lobata* (Willd.) Ohwi, Radix), Niuxi (*Achyranthes bidentata* Blume, Radix), Huanglian (*Coptis chinensis* Franch., Rhizoma), Danshen (*Salvia miltiorrhiza* Bunge, Radix et Rhizoma), Chuanxuduan (*Dipsacus asper* Wall. ex Henry, Radix), Yimucao (*Leonurus japonicus* Houtt., Herba) (doses not reported)
Gao 2012 (22)	Rougan Xifeng Tongluo Decoction	RGXFTL	Blood stasis; qi deficiency	Baishao (*Paeonia lactiflora* Pall., Radix) 20 g, Tianma (*Gastrodia elata* Blume, Rhizoma) 15 g, Danggui (*Angelica sinensis* (Oliv.) Diels, Radix) 10 g, Chuanxiong (*Ligusticum chuanxiong* Hort., Rhizoma) 10 g, Jiangcan (*Bombyx mori* L. infected with *Beauveria bassiana*, whole body) 10 g, Sigualuo (*Luffa cylindrica* (L.) Roem., Fructus Retinervus) 10 g, Baijiezi (*Sinapis alba* L., Semen) 6 g, Danshen (*Salvia miltiorrhiza* Bunge, Radix et Rhizoma) 20 g, Sheng-gancao (*Glycyrrhiza uralensis* Fisch., Radix et Rhizoma) 6 g
Zhou 2014 (23)	Xiaoma Zhitong Capsule	XMZT	Blood stasis with obstruction of the collaterals; yin deficiency	Huangqi (*Astragalus membranaceus* Fisch. ex Bunge, Radix) 30 g; Shudihuang (*Rehmannia glutinosa* Libosch., Radix Preparata), Danggui (*Angelica sinensis* (Oliv.) Diels, Radix), Chuanxiong (*Ligusticum chuanxiong* Hort., Rhizoma), Baishao (*Paeonia lactiflora* Pall., Radix), Yanhusuo (*Corydalis yanhusuo* W.T. Wang, Rhizoma), Jixueteng (*Spatholobus suberectus* Dunn, Caulis), Luoshiteng (*Trachelospermum jasminoides* (Lindl.) Lem., Caulis et Folium), Daxueteng (*Sargentodoxa cuneata* (Oliv.) Rehd. et Wils., Caulis), Weilingxian (*Clematis chinensis* Osbeck, Radix et Rhizoma), Yinyanghuo (*Epimedium brevicornu* Maxim., Folium), Luxiancao (*Pyrola calliantha* H. Andres, Herba), Xixiancao (*Siegesbeckia orientalis* L., Herba) and Danshen (*Salvia miltiorrhiza* Bunge, Radix et Rhizoma), 10 g each; Tianlong (*Gekko japonicus* Dumeril et Bibron, whole body) and Dilong (*Pheretima aspergillum* (E. Perrier), whole body), 5 g each
Song 2015 (24)	Modified Guizhi Shaoyao Zhimu Decoction	JWGSZM	Blood stasis; obstruction of the collaterals	Guizhi (*Cinnamomum cassia* Presl, Ramulus) 10 g, Baishao (*Paeonia lactiflora* Pall., Radix) 15 g, Mahuang (*Ephedra sinica* Stapf, Herba) 5 g, Baizhu (*Atractylodes macrocephala* Koidz., Rhizoma) 15 g, Zhimu (*Anemarrhena asphodeloides* Bunge, Rhizoma) 15 g, Fangfeng (*Saposhnikovia divaricata* (Turcz.) Schischk., Radix) 10 g, Shu-fuzi (*Aconitum carmichaelii* Debx., Radix Lateralis Preparata) 10–15 g, Ganjiang (*Zingiber officinale* Rosc., Rhizoma) 10 g, Gancao (*Glycyrrhiza uralensis* Fisch., Radix et Rhizoma) 10 g, Jixueteng (*Spatholobus suberectus* Dunn, Caulis) 30 g
Liu 2015 (25)	Modified Huangqi Guizhi Wuwu Decoction	JWHQGZ	Blood stasis; obstruction of the collaterals	Huangqi (*Astragalus membranaceus* Fisch. ex Bunge, Radix) 60 g, Guizhi (*Cinnamomum cassia* Presl, Ramulus) 15 g, Chishao (*Paeonia lactiflora* Pall., Radix) 15 g, Danshen (*Salvia miltiorrhiza* Bunge, Radix et Rhizoma) 15 g, Danggui (*Angelica sinensis* (Oliv.) Diels, Radix) 15 g, Dilong (*Pheretima aspergillum* (E. Perrier), whole body) 15 g, Chuanxiong (*Ligusticum chuanxiong* Hort., Rhizoma) 15 g, Shengjiang (*Zingiber officinale* Rosc., Rhizoma Recens) 15 g, Dazao (*Ziziphus jujuba* Mill., Fructus) 15 g
Wang 2017 (26)	Danggui Sini Decoction	DGSN	Blood stasis; obstruction of the collaterals	Guizhi (*Cinnamomum cassia* Presl, Ramulus) 15 g, Xixin (*Asarum heterotropoides* Fr. Schmidt var. *mandshuricum* (Maxim.) Kitag., Radix et Rhizoma) 5 g, Danggui (*Angelica sinensis* (Oliv.) Diels, Radix) 15 g, Zhi-gancao (*Glycyrrhiza uralensis* Fisch., Radix et Rhizoma, honey-fried) 10 g, Baishao (*Paeonia lactiflora* Pall., Radix) 15 g, Dazao (*Ziziphus jujuba* Mill., Fructus) 10 g, Tongcao (*Tetrapanax papyrifer* (Hook.) K. Koch, Medulla) 10 g
Fang 2017 (27)	Yiqi Huoxue Formula	YQHX	Blood stasis; obstruction of the collaterals	Huangqi (*Astragalus membranaceus* Fisch. ex Bunge, Radix) 30 g, Chishao (*Paeonia lactiflora* Pall., Radix) 15 g, Danggui-wei (*Angelica sinensis* (Oliv.) Diels, Radix, tail portion) 10 g, Honghua (*Carthamus tinctorius* L., Flos) 10 g, Moyao (*Commiphora myrrha* Engl., Resina) 10 g, Shudi (*Rehmannia glutinosa* Libosch., Radix Preparata) 10 g, Shanyao (*Dioscorea opposita* Thunb., Rhizoma) 10 g, Yiyiren (*Coix lacryma-jobi* L. var. *ma-yuen* (Roman.) Stapf, Semen) 10 g, Dilong (*Pheretima aspergillum* (E. Perrier), whole body) 3 g, Chuanxiong (*Ligusticum chuanxiong* Hort., Rhizoma) 6 g, Gancao (*Glycyrrhiza uralensis* Fisch., Radix et Rhizoma) 3 g, Chao-maiya (*Hordeum vulgare* L., Fructus Germinatus, stir-fried) 15 g, Chao-guya (*Setaria italica* (L.) Beauv., Fructus Germinatus, stir-fried) 15 g
Zhang 2018 (28)	Jiangtang Tongluo Tablet	JTT	Qi deficiency with blood stasis; obstruction of the collaterals	Huangqi (*Astragalus membranaceus* Fisch. ex Bunge, Radix) 50 g, Chuanxiong (*Ligusticum chuanxiong* Hort., Rhizoma) 12 g, Danggui (*Angelica sinensis* (Oliv.) Diels, Radix) 15 g, Dihuang (*Rehmannia glutinosa* Libosch., Radix) 15 g, Dilong (*Pheretima aspergillum* (E. Perrier), whole body) 30 g, Chishao (*Paeonia lactiflora* Pall., Radix) 30 g, Honghua (*Carthamus tinctorius* L., Flos) 30 g, Taoren (*Prunus persica* (L.) Batsch, Semen) 15 g, Quanxie (*Buthus martensii* Karsch, whole body) 10 g, Jixueteng (*Spatholobus suberectus* Dunn, Caulis) 30 g, Guijianyu (*Euonymus alatus* (Thunb.) Sieb., Ramulus alatus) 30 g
Pei 2019 (29)	Modified Buyang Huanwu Decoction	JWBYHW	Blood stasis; obstruction of the collaterals	Huangqi-pian (*Astragalus membranaceus* Fisch. ex Bunge, Radix, sliced) 30 g, Chishao (*Paeonia lactiflora* Pall., Radix) 15 g, Danggui (*Angelica sinensis* (Oliv.) Diels, Radix) 12 g, Taoren (*Prunus persica* (L.) Batsch, Semen) 12 g, Dilong (*Pheretima aspergillum* (E. Perrier), whole body) 9 g, Chuanxiong (*Ligusticum chuanxiong* Hort., Rhizoma) 9 g, Xihonghua (*Crocus sativus* L., Stigma) 6 g, Jixueteng (*Spatholobus suberectus* Dunn, Caulis) 15 g, Tianma (*Gastrodia elata* Blume, Rhizoma) 9 g, Jiangcan (*Bombyx mori* L. infected with *Beauveria bassiana*, whole body) 9 g, Tubiechong (*Eupolyphaga sinensis* Walker, whole body) 3 g, Shuizhi (*Whitmania pigra* Whitman, whole body) 3 g, Qianglang (*Catharsius molossus* L., whole body) 3 g
Zhao 2019 (30)	Danggui Sini Decoction	DGSN	Blood stasis; obstruction of the collaterals	Danggui (*Angelica sinensis* (Oliv.) Diels, Radix) 18 g, Guizhi (*Cinnamomum cassia* Presl, Ramulus) 12 g, Huangqi (*Astragalus membranaceus* Fisch. ex Bunge, Radix) 15 g, Shaoyao (*Paeonia lactiflora* Pall., Radix) 15 g, Xixin (*Asarum heterotropoides* Fr. Schmidt var. *mandshuricum* (Maxim.) Kitag., Radix et Rhizoma) 3 g, Zhi-gancao (*Glycyrrhiza uralensis* Fisch., Radix et Rhizoma, honey-fried) 6 g, Tongcao (*Tetrapanax papyrifer* (Hook.) K. Koch, Medulla) 12 g
Zhi 2020 (31)	Huoluo Zhixiao Formula	HLZX	Blood stasis with obstruction of the collaterals; qi deficiency	Gouji (*Cibotium barometz* (L.) J. Sm., Rhizoma) 10 g, Xuduan (*Dipsacus asper* Wall. ex Henry, Radix) 10 g, Chuanxiong (*Ligusticum chuanxiong* Hort., Rhizoma) 10 g, Danshen (*Salvia miltiorrhiza* Bunge, Radix et Rhizoma) 15 g, Niuxi (*Achyranthes bidentata* Blume, Radix) 15 g, Guijianyu (*Euonymus alatus* (Thunb.) Sieb., Ramulus alatus) 10 g, Mugua (*Chaenomeles speciosa* (Sweet) Nakai, Fructus) 15 g, Tubiechong (*Eupolyphaga sinensis* Walker, whole body) 5 g, Shuizhi (*Whitmania pigra* Whitman, whole body) 3 g, Wugong (*Scolopendra subspinipes mutilans* L. Koch, whole body) 2 pieces, Sheng-gancao (*Glycyrrhiza uralensis* Fisch., Radix et Rhizoma) 3 g
Bao 2020 (32)	Yiqi Tongluo Huoxue Decoction	YQTLHX	Qi deficiency with blood stasis	Huangqi (*Astragalus membranaceus* Fisch. ex Bunge, Radix) 15 g, Taoren (*Prunus persica* (L.) Batsch, Semen) 10 g, Danggui (*Angelica sinensis* (Oliv.) Diels, Radix) 10 g, Danshen (*Salvia miltiorrhiza* Bunge, Radix et Rhizoma) 10 g, Shengdihuang (*Rehmannia glutinosa* Libosch., Radix) 10 g, Sanqi (*Panax notoginseng* (Burk.) F.H. Chen, Radix et Rhizoma) 10 g, Jixueteng (*Spatholobus suberectus* Dunn, Caulis) 10 g, Chuan-niuxi (*Cyathula officinalis* Kuan, Radix) 10 g, Maidong (*Ophiopogon japonicus* (L. f.) Ker-Gawl., Radix) 6 g, Digupi (*Lycium chinense* Mill., Cortex Radicis) 6 g, Dilong (*Pheretima aspergillum* (E. Perrier), whole body) 6 g, Gancao (*Glycyrrhiza uralensis* Fisch., Radix et Rhizoma) 10 g
Huang 2021 (33)	Buqi Tongluo Capsule	BQTL	Blood stasis with obstruction of the vessels and collaterals; qi deficiency	Danshen (*Salvia miltiorrhiza* Bunge, Radix et Rhizoma) 714 g, Chuanxiong (*Ligusticum chuanxiong* Hort., Rhizoma) 595 g, Danggui (*Angelica sinensis* (Oliv.) Diels, Radix) 714 g, Huangqi (*Astragalus membranaceus* Fisch. ex Bunge, Radix) 893 g, Hongshen (*Panax ginseng* C. A. Mey., Radix et Rhizoma Rubra) 298 g, Jianghuang (*Curcuma longa* L., Rhizoma) 714 g, with appropriate excipients to make 1000 capsules
Zhu 2023 (34)	Shengyang Sanhuo Decoction	SYSH	Blood stasis with obstruction of the collaterals; qi stagnation	Shengma (*Cimicifuga heracleifolia* Kom., Rhizoma) 15 g, Huangqi (*Astragalus membranaceus* Fisch. ex Bunge, Radix) 30 g, Gegen (*Pueraria lobata* (Willd.) Ohwi, Radix) 15 g, Qianghuo (*Notopterygium incisum* Ting ex H. T. Chang, Rhizoma et Radix) 15 g, Duhuo (*Angelica pubescens* Maxim. f. *biserrata* Shan et Yuan, Radix) 15 g, Fangfeng (*Saposhnikovia divaricata* (Turcz.) Schischk., Radix) 9 g, Chaihu (*Bupleurum chinense* DC., Radix) 9 g, Baishao (*Paeonia lactiflora* Pall., Radix) 15 g, Danggui (*Angelica sinensis* (Oliv.) Diels, Radix) 15 g, Danshen (*Salvia miltiorrhiza* Bunge, Radix et Rhizoma) 15 g, Renshen (*Panax ginseng* C. A. Mey., Radix et Rhizoma) 15 g, Zhi-gancao (*Glycyrrhiza uralensis* Fisch., Radix et Rhizoma, honey-fried) 6 g, Sheng-gancao (*Glycyrrhiza uralensis* Fisch., Radix et Rhizoma) 6 g
Lei 2024 (35)	Yiqi Huashi Tongbi Formula	YQHSTB	Blood stasis with obstruction of the vessels and collaterals; qi deficiency	Huangqi (*Astragalus membranaceus* Fisch. ex Bunge, Radix) 15 g, Guizhi (*Cinnamomum cassia* Presl, Ramulus) 10 g, Baishao (*Paeonia lactiflora* Pall., Radix) 15 g, Shengjiang (*Zingiber officinale* Rosc., Rhizoma Recens) 15 g, Dazao (*Ziziphus jujuba* Mill., Fructus) 20 g, Danggui (*Angelica sinensis* (Oliv.) Diels, Radix) 10 g, Chuanxiong (*Ligusticum chuanxiong* Hort., Rhizoma) 10 g, Fuling (*Poria cocos* (Schw.) Wolf, Sclerotium) 15 g, Cangzhu (*Atractylodes lancea* (Thunb.) DC., Rhizoma) 10 g, Zexie (*Alisma orientale* (Sam.) Juzep., Rhizoma) 15 g, Jixueteng (*Spatholobus suberectus* Dunn, Caulis) 20 g, Dilong (*Pheretima aspergillum* (E. Perrier), whole body) 10 g
Jia 2025 (36)	Huangqi Guizhi Wuwu Decoction	HQGZWW	Blood stasis; obstruction of the collaterals; qi deficiency	Huangqi (*Astragalus membranaceus* Fisch. ex Bunge, Radix) 40 g, Guizhi (*Cinnamomum cassia* Presl, Ramulus) 15 g, Jixueteng (*Spatholobus suberectus* Dunn, Caulis) 15 g, Baishao (*Paeonia lactiflora* Pall., Radix) 15 g, Honghua (*Carthamus tinctorius* L., Flos) 10 g, Niuxi (*Achyranthes bidentata* Blume, Radix) 10 g, Ruxiang (*Boswellia carterii* Birdw., Resina) 10 g, Shengjiang (*Zingiber officinale* Rosc., Rhizoma Recens) 10 g, Gancao (*Glycyrrhiza uralensis* Fisch., Radix et Rhizoma) 6 g, Dazao (*Ziziphus jujuba* Mill., Fructus) 3 pieces
Deng 2025 (37)	Modified Buyang Huanwu Decoction	JWBYHW	Qi deficiency; obstruction of the collaterals; blood stasis	Sheng-huangqi (*Astragalus membranaceus* Fisch. ex Bunge, Radix) 30 g, Niuxi (*Achyranthes bidentata* Blume, Radix) 30 g, Taizishen (*Pseudostellaria heterophylla* (Miq.) Pax ex Pax et Hoffm., Radix) 15 g, Huai-shanyao (*Dioscorea opposita* Thunb., Rhizoma) 15 g, Chishao (*Paeonia lactiflora* Pall., Radix) 15 g, Jinyinhua (*Lonicera japonica* Thunb., Flos) 15 g, Tianhuafen (*Trichosanthes kirilowii* Maxim., Radix) 15 g, Zihuadiding (*Viola yedoensis* Makino, Herba) 15 g, Chao-baishao (*Paeonia lactiflora* Pall., Radix, stir-fried) 15 g, Danggui-wei (*Angelica sinensis* (Oliv.) Diels, Radix, tail portion) 10 g, Chuanxiong (*Ligusticum chuanxiong* Hort., Rhizoma) 10 g, Danshen (*Salvia miltiorrhiza* Bunge, Radix et Rhizoma) 15 g, Honghua (*Carthamus tinctorius* L., Flos) 6 g, Dilong (*Pheretima aspergillum* (E. Perrier), whole body) 10 g, Zhi-gancao (*Glycyrrhiza uralensis* Fisch., Radix et Rhizoma, honey-fried) 6 g
Zhao 2025 (38)	TangBi Formula	TBF	Blood stasis; obstruction of the collaterals	Huangqi (*Astragalus membranaceus* Fisch. ex Bunge, Radix), Guizhi (*Cinnamomum cassia* Presl, Ramulus), Chuanxiong (*Ligusticum chuanxiong* Hort., Rhizoma), Baishao (*Paeonia lactiflora* Pall., Radix), Jixueteng (*Spatholobus suberectus* Dunn, Caulis) (doses not reported)
Jin 2026 (39)	Mudan Granule	MDKL	Blood stasis; obstruction of the collaterals; qi deficiency	Huangqi (*Astragalus membranaceus* Fisch. ex Bunge, Radix), Yanhusuo (*Corydalis yanhusuo* W.T. Wang, Rhizoma), Sanqi (*Panax notoginseng* (Burk.) F.H. Chen, Radix et Rhizoma), Chishao (*Paeonia lactiflora* Pall., Radix), Danshen (*Salvia miltiorrhiza* Bunge, Radix et Rhizoma), Chuanxiong (*Ligusticum chuanxiong* Hort., Rhizoma), Honghua (*Carthamus tinctorius* L., Flos), Sumu (*Caesalpinia sappan* L., Lignum), Jixueteng (*Spatholobus suberectus* Dunn, Caulis) (doses not reported)

**Table 1b:** Preparation, administration, comparator regimen, background therapy and diagnostic criteria.

Study	Preparation and administration	Treatment duration, weeks	Mecobalamin regimen in the control arm	Background therapy	Diagnostic criteria
Shen 2009 (21)	Capsule, 4.5 g of crude drug per capsule; 5 capsules per dose, twice daily, orally	8	Mecobalamin 0.5 mg three times daily, orally	Routine glucose-lowering therapy plus blood pressure and lipid control	WHO 1999 criteria plus Practical Endocrinology
Gao 2012 (22)	Decoction, one dose daily, taken orally in two divided portions	8	Mecobalamin 0.5 mg three times daily, orally	Routine glucose-lowering therapy plus blood pressure and lipid control as required	WHO 1999 criteria plus Diagnosis and Treatment of Diabetes and Its Complications in Integrated Chinese and Western Medicine (1997)
Zhou 2014 (23)	In-hospital capsule preparation, 100 capsules per bottle; 4 capsules per dose, three times daily, orally	12	Mecobalamin 500 ug three times daily, orally; plus 4 starch placebo capsules per dose, three times daily	Routine glucose-lowering therapy, exercise and diet	China Guideline for the Prevention and Treatment of Diabetes (2013)
Song 2015 (24)	Decoction, decocted twice with 200 mL obtained each time, taken in the morning and evening	12	Mecobalamin 0.5 mg three times daily, orally	Routine glucose- and blood pressure-lowering therapy plus blood pressure and lipid control as required	China Guideline for the Prevention and Treatment of Diabetes (2013)
Liu 2015 (25)	Decoction, one dose daily	8	Mecobalamin 0.5 mg three times daily, orally	Routine glucose-lowering therapy plus blood pressure and lipid control as required	WHO 1999 criteria plus Chinese Guideline for the Prevention and Treatment of Type 2 Diabetes (2010)
Wang 2017 (26)	Decoction, concentrated under pressure to 200 mL; 200 mL per dose, twice daily, orally	8	Mecobalamin 500 ug three times daily, orally	Routine glucose-lowering therapy, exercise and diet	ADA 1997 criteria plus Integrated Chinese and Western Medicine Treatment of Diabetes (2002)
Fang 2017 (27)	Decoction, one dose daily, three times daily, taken 30 min after meals	8	Mecobalamin 0.5 mg three times daily, orally	Routine glucose-lowering therapy, HbA1c below 7.5%	Chinese Guideline for the Prevention and Treatment of Type 2 Diabetes (2017)
Zhang 2018 (28)	In-hospital tablet preparation (Henan preparation approval no. Z04020181), 2.5 g of crude drug per tablet; 3 tablets per dose, three times daily, orally	8	Mecobalamin 500 ug three times daily, orally	Routine glucose-lowering therapy, fasting plasma glucose 4.4-7.0 mmol/L	Chinese Guideline for the Prevention and Treatment of Type 2 Diabetes (2013)
Pei 2019 (29)	Decoction, soaked in cold water for 60 min then decocted for 60 min to yield 400 mL; 200 mL per dose, taken in the morning and evening	4	Mecobalamin 0.5 mg three times daily, orally	Medical nutrition therapy plus glycaemic control	Chinese Guideline for the Prevention and Treatment of Type 2 Diabetes (2013)
Zhao 2019 (30)	Decoction, one dose daily, taken in two divided portions	4	Mecobalamin 500 ug three times daily, orally	Routine glucose-lowering therapy, diabetes education and dietary control	Chinese Guideline for the Prevention and Treatment of Type 2 Diabetes (2013)
Zhi 2020 (31)	Decoction, prepared with 1200 mL of water, taken in two divided portions in the morning and evening	20	Mecobalamin 0.5 mg three times daily, orally	Routine glucose-lowering therapy (biguanide, sulfonylurea, alpha-glucosidase inhibitor or insulin)	Chinese Guideline for the Prevention and Treatment of Type 2 Diabetes (2017)
Bao 2020 (32)	Decoction, decocted to 400 mL, one dose daily, twice daily	8	Mecobalamin 0.5 mg three times daily, orally	Blood pressure and glycaemic control, fasting plasma glucose below 7.0 mmol/L	Chinese Guideline for the Prevention and Treatment of Type 2 Diabetes (2017)
Huang 2021 (33)	In-hospital capsule preparation; 3 capsules per dose, three times daily, orally	4	Mecobalamin 0.5 mg three times daily, orally	Routine glucose-lowering therapy, exercise and diet	Chinese Medicine Clinical Guideline for the Diagnosis and Treatment of Diabetic Peripheral Neuropathy (2016)
Zhu 2023 (34)	Decoction, one dose daily, taken warm after breakfast and dinner	12	Mecobalamin 0.5 mg three times daily, orally	Diet and exercise, glucose-lowering therapy and blood pressure control	Chinese Guideline for the Prevention and Treatment of Type 2 Diabetes (2020)
Lei 2024 (35)	Granules, dissolved in 300 mL of boiling water, taken in two divided portions in the morning and evening, one dose daily	8	Mecobalamin 500 ug three times daily, orally	Diet and exercise, blood pressure and glycaemic control	Chinese Guideline for the Prevention and Treatment of Type 2 Diabetes (2013)
Jia 2025 (36)	Decoction, 1200 mL of water decocted to 400 mL, taken warm in three divided portions	12	Mecobalamin 0.5 mg three times daily, orally	Diet and exercise, glucose-lowering and blood pressure-lowering therapy, vitamin B1	Chinese Guideline for the Prevention and Treatment of Type 2 Diabetes (2020) plus San Antonio Consensus 1988
Deng 2025 (37)	Decoction, one dose daily, decocted to 300 mL, taken warm in two divided portions in the morning and evening	12	Mecobalamin 0.5 mg three times daily, orally	Lifestyle and dietary adjustment plus glycaemic control	Chinese Guideline for the Prevention and Treatment of Type 2 Diabetes (2017)
Zhao 2025 (38)	Granules, 6 g per sachet, twice daily, orally	24	Mecobalamin tablets 500 ug three times daily, orally (plus placebo granules)	Diabetes education, diet and glycaemic control	Chinese Guideline for the Prevention and Treatment of Type 2 Diabetes (2017)
Jin 2026 (39)	Marketed Chinese patent medicine (State approval no. Z20080033), 7 g per sachet; 7 g per dose, three times daily, taken orally after meals	24	Mecobalamin 0.5 mg three times daily, orally after meals	Lifestyle intervention plus the baseline glucose-lowering regimen, HbA1c below 8.5%	Chinese Guideline for the Prevention and Treatment of Type 2 Diabetes (2017)

### 2.4 Outcomes

The primary efficacy outcome was the TCSS, a composite measure of the symptoms, signs and severity of nerve injury in DPN (18). Secondary efficacy outcomes were motor (MCV) and sensory (SCV) conduction velocity of the median and common peroneal nerves. A further secondary outcome was the incidence of adverse events (AEs); AE information was extracted from the safety reporting of each trial, with particular attention to hepatic function, renal function, allergic reactions, hypoglycaemia, gastrointestinal intolerance and potential formula-drug interactions.

### 2.5 Search strategy

The following databases were searched systematically: PubMed, Embase, Web of Science, the Cochrane Library, China National Knowledge Infrastructure (CNKI), Wanfang Data and the VIP Chinese Science and Technology Journal Database. Each database was searched from inception to 31 July 2026, and records were restricted to reports in Chinese and English. The strategies combined medical subject headings (MeSH/Emtree), Chinese subject headings and free-text terms, adapted to the syntax of each database; the core concepts were a disease block and an intervention block, combined with Boolean operators. Full search strategies are given in **Supplementary Table 2**.

### 2.6 Study selection and data extraction

Study selection followed a standardised, blinded two-reviewer procedure designed to reduce selection bias. After deduplication, two reviewers independently screened titles and abstracts against the pre-specified PICOS criteria and excluded records that were clearly ineligible. Exclusion criteria were defined strictly as: (1) non-randomised designs, including observational cohorts, case series, retrospective studies and before-after comparisons; (2) intervention groups receiving non-pharmacological therapy containing no traditional Chinese medicine component, such as acupuncture, tuina, acupoint application or gua sha alone; (3) duplicate publication or overlapping data sources; (4) incomplete core outcome data that could not be obtained from the authors; and (5) basic experiments in animals or cells, reviews, case reports and summaries of clinical experience. Records passing the initial screen underwent full-text assessment to determine final eligibility. Disagreements were resolved by discussion, and any that remained were adjudicated by a third reviewer. The complete selection process is shown in the PRISMA flow diagram (**Figure 1**).

**Figure 1:**
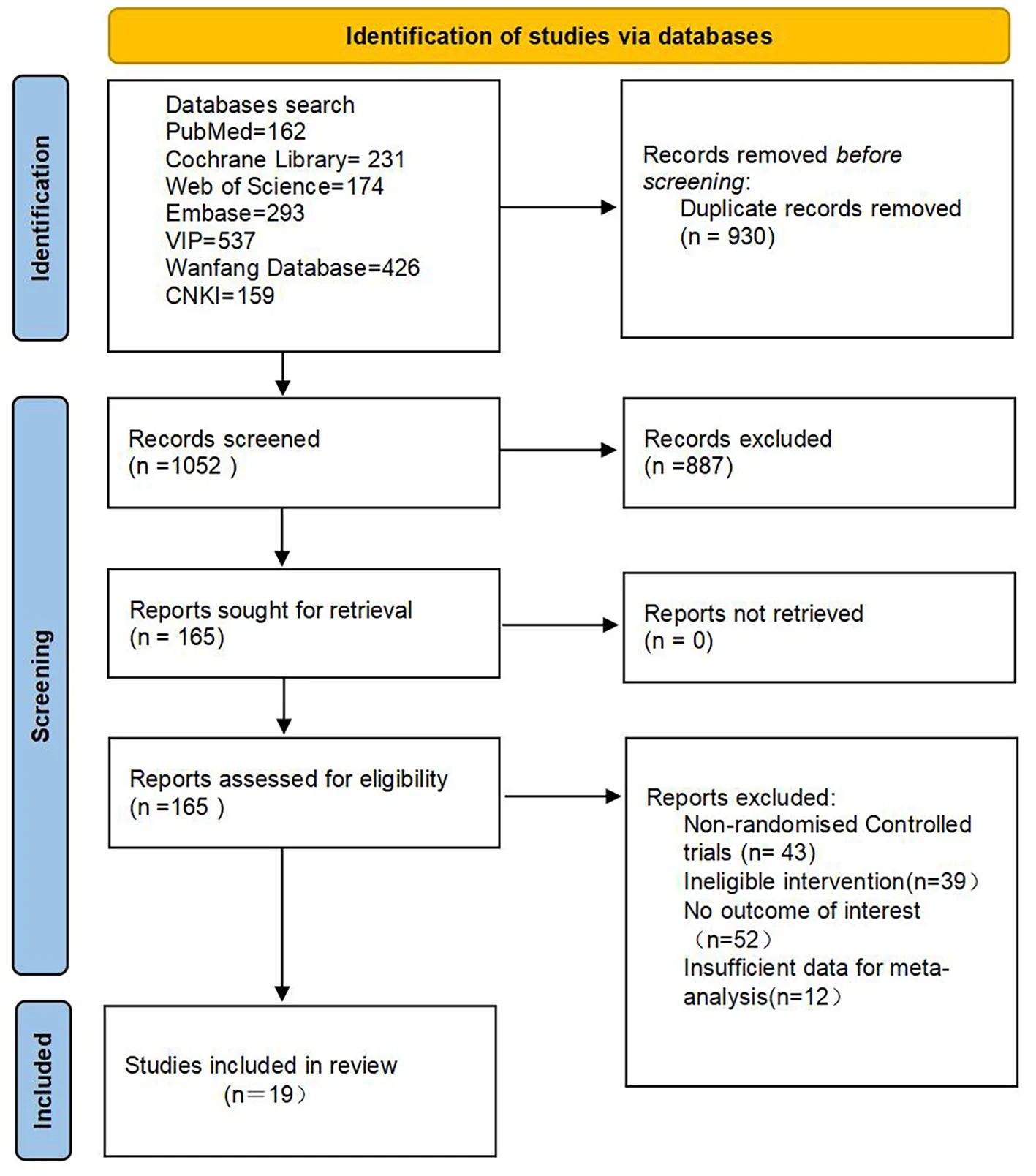
PRISMA flow diagram.

Data were extracted independently by two reviewers using a standardised form calibrated in a pilot exercise, covering three domains: (1) study characteristics, namely first author, year of publication, study design, sample size in each arm and treatment duration; (2) clinical and demographic information, namely mean age, sex distribution, diagnostic criteria for diabetes and DPN, disease duration and baseline neurophysiological parameters; and (3) details of the intervention and comparator, namely formula name and dosage form, full composition, route and frequency of administration, background treatment in the control arm, timing of outcome measurement and adverse events. For continuous outcomes, the mean difference (MD) from baseline, the standard deviation (SD) and the sample size (n) were extracted. Where a primary report gave only the standard error, a confidence interval, a P value or an interquartile range, algebraic conversion and variance estimation were performed using the methods recommended in the Cochrane Handbook (19). Change scores served as the principal analytical quantity. All extracted data were cross-checked item by item by two reviewers before pooling, and disagreements were resolved by rechecking the source article, by discussion or by third-party adjudication. Where the SD of the change score was not directly available, it was derived from the pre- and post-treatment SDs assuming a correlation coefficient of r = 0.7; a sensitivity analysis across r = 0.5 to 0.9 is given in **Supplementary Table 8**. When a trial reported several post-treatment time points, data from the end of the treatment course were extracted throughout.

### 2.7 Risk of bias and certainty of evidence

At the level of the individual study, methodological quality was assessed with version 2 of the Cochrane risk-of-bias tool (RoB 2) across five domains: the randomisation process, deviations from intended interventions, missing outcome data, measurement of the outcome, and selection of the reported result. The certainty of evidence for every network estimate was assessed with the CINeMA framework (version 2.0.0), which covers six domains: within-study bias, reporting bias, indirectness, imprecision, heterogeneity and incoherence. Risk-of-bias assessment and certainty rating were carried out independently by two reviewers, with disagreements resolved by discussion or third-party adjudication.

### 2.8 Statistical analysis

Analyses were performed in R (version 4.4.1). Effect estimates were interpreted in the light of clinical plausibility, network geometry, the risk-of-bias profile of the contributing studies and the certainty of evidence.

#### 2.8.1 Pairwise meta-analysis

Continuous outcomes were expressed as MDs with 95% confidence intervals. A random-effects model was fitted using REML estimation with the Hartung-Knapp adjustment, and confidence intervals and P values were derived from the t distribution (df = k - 1); tests of effect and of heterogeneity were reported separately. Prediction intervals were also calculated. Outcome direction was harmonised before pooling: for TCSS a fall in score denotes improvement (negative MD), whereas for nerve conduction velocity a rise denotes improvement (positive MD). These pooled results served as an empirical reference for the scale of heterogeneity and as the anchor for the Bayesian priors.

#### 2.8.2 Bayesian network meta-analysis

The NMA was implemented within a Bayesian hierarchical modelling framework using the R package gemtc (version 1.0.2) through the rjags interface (version 4.16), with JAGS 4.3.1 as the backend. The model used a normal likelihood with an identity link function, the mean difference as the effect measure and mecobalamin as the network reference treatment. Study-specific baseline effects and basic parameters were assigned normal priors with mean 0 and variance 10^4.

The primary analysis used a random-effects consistency model in which the between-study standard deviation was given a half-normal prior whose scale was taken from the REML estimate of the between-study standard deviation in the pairwise meta-analysis of the same data, subject to a floor of 0.5. Sensitivity analyses comprised a common-effect model, a uniform-prior model with the upper bound set to three times that estimate, subject to a floor of 2, and models fixing the between-study standard deviation at 0, 0.5, 1.0 and 1.5 times that estimate. The MCMC scheme used 4 parallel chains with 20,000 adaptation iterations, followed by 100,000 iterations per chain thinned at an interval of 10, retaining 40,000 posterior samples in total. Convergence was assessed with the Gelman-Rubin potential scale reduction factor (threshold 1.05), the effective sample size and the Monte Carlo standard error, supported by inspection of trace and density plots. Model fit was evaluated with the residual deviance per data point.

#### 2.8.3 Assessment of consistency

All evidence networks in this study were star-shaped: each combination regimen formed a direct comparison only with the common comparator, mecobalamin, and every pairwise comparison between formulas therefore rested on indirect evidence. None of the five outcome networks contained a closed loop, so neither node-splitting nor global tests of consistency were applicable, and inconsistency could not be assessed statistically in this review.

#### 2.8.4 Treatment ranking

The relative ranking of interventions is presented as the surface under the cumulative ranking curve (SUCRA) (20). SUCRA summarises cumulative ranking probabilities on a scale from 0% to 100%. Given the sparse network, the high proportion of small trials and the fact that most nodes are informed by a single trial, the uncertainty of the effect estimates is not conveyed by the ranking metric. Rankings are therefore presented as exploratory and reported alongside the effect estimates and their credible intervals.

#### 2.8.5 Sensitivity analyses and small-study effects

To evaluate the potential influence of any single study on the network estimates, we carried out a leave-one-out sensitivity analysis at the network level. It should be noted at the outset that in a star-shaped network the removal of a study also removes the node to which it belongs; the analysis can therefore examine only the relative ranking of the remaining nodes and cannot assess the robustness of the effect estimate for the omitted node itself. The results of this analysis are treated as descriptive and are not used as the principal basis for inference about the NMA rankings.

Small-study effects and publication bias were examined with Egger’s regression, the Begg-Mazumdar rank correlation and the Thompson-Sharp test, all using the same random-effects model as the primary analysis. Comparison-adjusted funnel plots could not be used because most comparisons contained only a single study (**Supplementary Table 4**), so category-level funnel plots are presented instead (**Figure 2**). For treatment nodes formed from two or more studies, the node was additionally split into separate nodes and the model refitted, with rankings compared before and after splitting.

**Figure 2:**
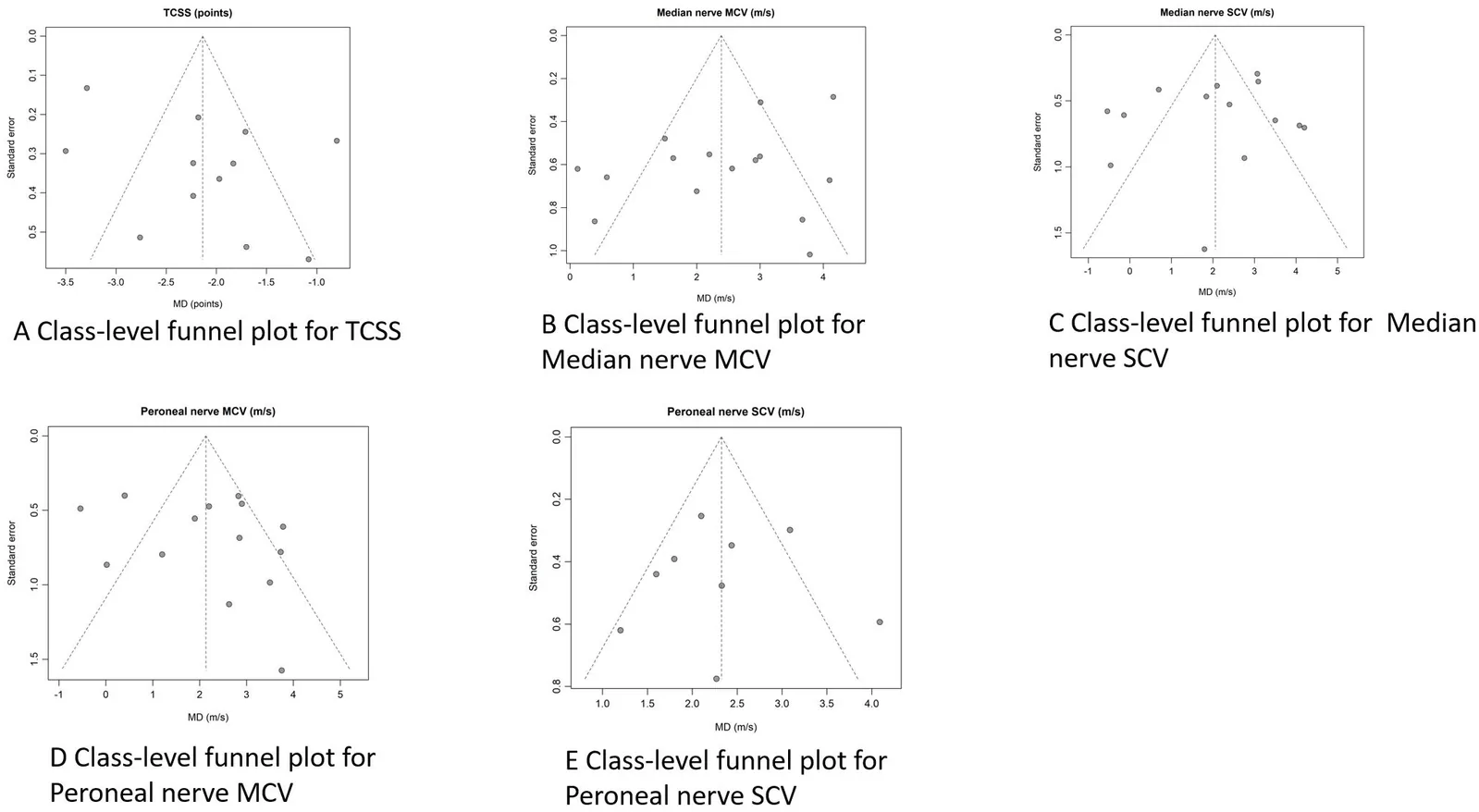
Risk of bias in the included trials, assessed with the Cochrane risk-of-bias tool for randomised trials (RoB 2). **(A, C)** Subjective outcome (Toronto Clinical Scoring System; 12 trials). **(B, D)** Objective outcomes (nerve conduction velocity; 18 trials). **(A, B)** Traffic-light plots showing domain-level and overall judgements for each trial. **(C, D)** Summary plots showing the distribution of judgements across trials as percentages. D1, randomisation process; D2, deviations from the intended interventions; D3, missing outcome data; D4, measurement of the outcome; D5, selection of the reported result.

## 3 Results

### 3.1 Search results and study characteristics

Systematic searching and stepwise screening yielded 19 RCTs (21–39) comprising 2,090 participants, 1,046 in the intervention arms and 1,044 in the control arms. The selection process is shown in **Figure 1**.

Sample size ranged from 60 to 400 participants per trial, treatment duration from 4 to 24 weeks and mean age from 49.5 to 72.3 years. Characteristics of the included trials are given in **Supplementary Tables 3a, b**, and formula compositions in **Tables 1a**, **b**.

The distribution of effect modifiers across treatment nodes is shown in **Supplementary Tables 3c, d**. Coefficients of variation across nodes ranged from 6.7% to 14.6% for age, baseline TCSS and baseline nerve conduction velocity. Missing-data rates ranged from 42% to 89% for duration of diabetes, duration of DPN, glycated haemoglobin and fasting plasma glucose, so transitivity could not be adequately evaluated on these four dimensions.

### 3.2 Network meta-analysis

#### 3.2.1 Toronto clinical scoring system

Twelve studies (1,379 participants) reported TCSS, forming a star-shaped evidence network of 12 formulas and mecobalamin. The 95% CrI excluded 0 for 9 formulas. By SUCRA, Yiqi Huashi Tongbi Formula ranked first (90.3%), Huangqi Guizhi Wuwu Decoction second (86.7%) and Modified Buyang Huanwu Decoction third (73.5%), with mecobalamin last (2.9%). The MD was -3.49 (95% CrI -5.17 to -1.81) for Yiqi Huashi Tongbi Formula, -3.28 (-4.93 to -1.64) for Huangqi Guizhi Wuwu Decoction and -2.76 (-4.64 to -0.90) for Modified Buyang Huanwu Decoction. The 95% CrI included 0 for three formulas: Modified Guizhi Shaoyao Zhimu Decoction, Yiqi Huoxue Formula and TangBi Formula. Pairwise comparisons are shown in **Figure 3**.

**Figure 3:**
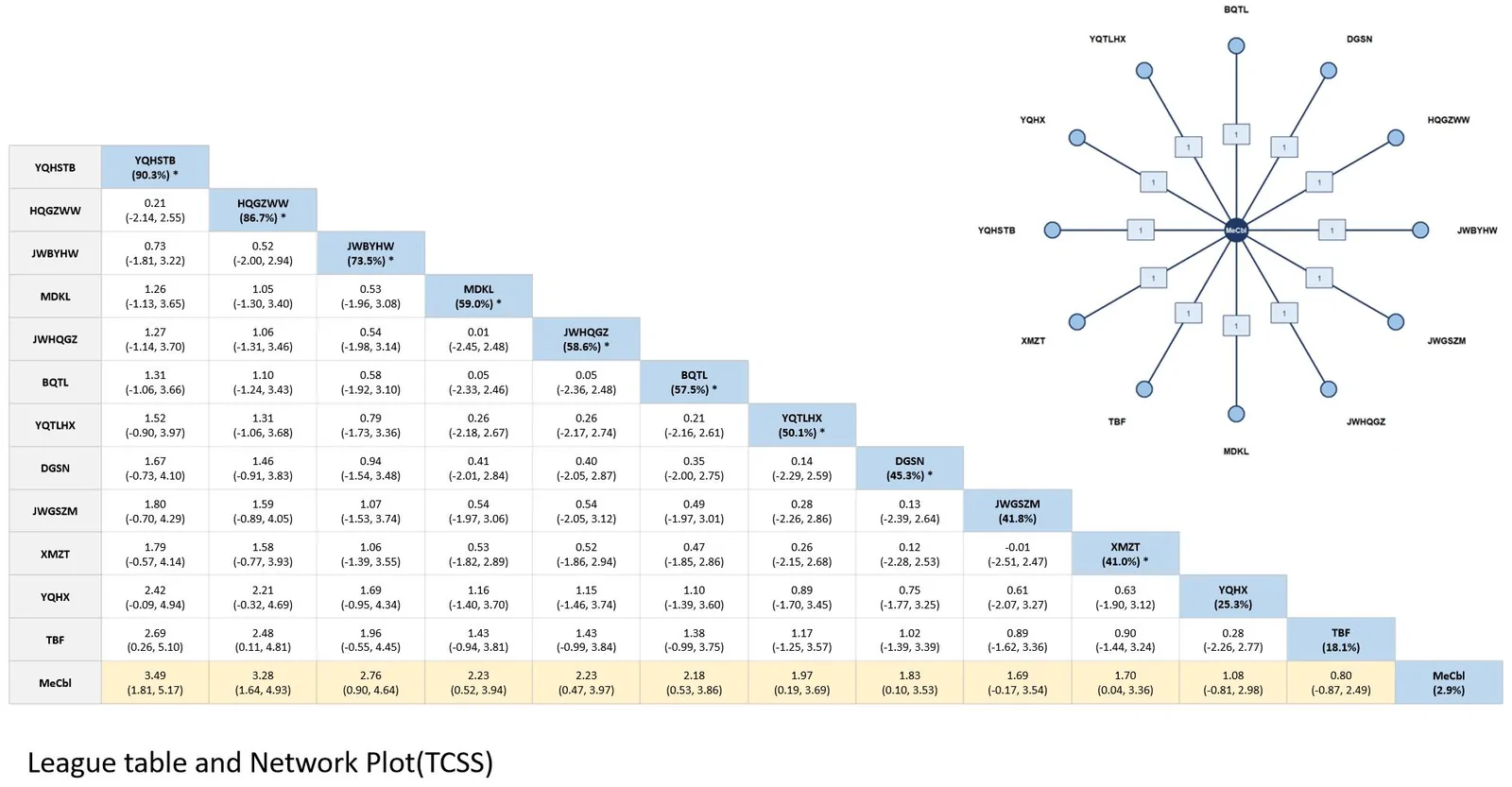
League table and network plot (TCSS).

#### 3.2.2 Median nerve motor conduction velocity

Fifteen studies (1,704 participants) reported median nerve MCV across 14 formulas. The 95% CrI excluded 0 for 7 formulas. By SUCRA, Shengyang Sanhuo Decoction ranked first (85.6%), Yiqi Huoxue Formula second (83.5%) and Modified Huangqi Guizhi Wuwu Decoction third (76.5%), with mecobalamin last (10.4%). The MD was 4.16 m/s (95% CrI 1.58 to 6.78) for Shengyang Sanhuo Decoction, 4.09 m/s (1.23 to 6.92) for Yiqi Huoxue Formula and 3.67 m/s (0.69 to 6.62) for Modified Huangqi Guizhi Wuwu Decoction. The 95% CrI included 0 for the remaining 7 formulas (**Figure 4**).

**Figure 4:**
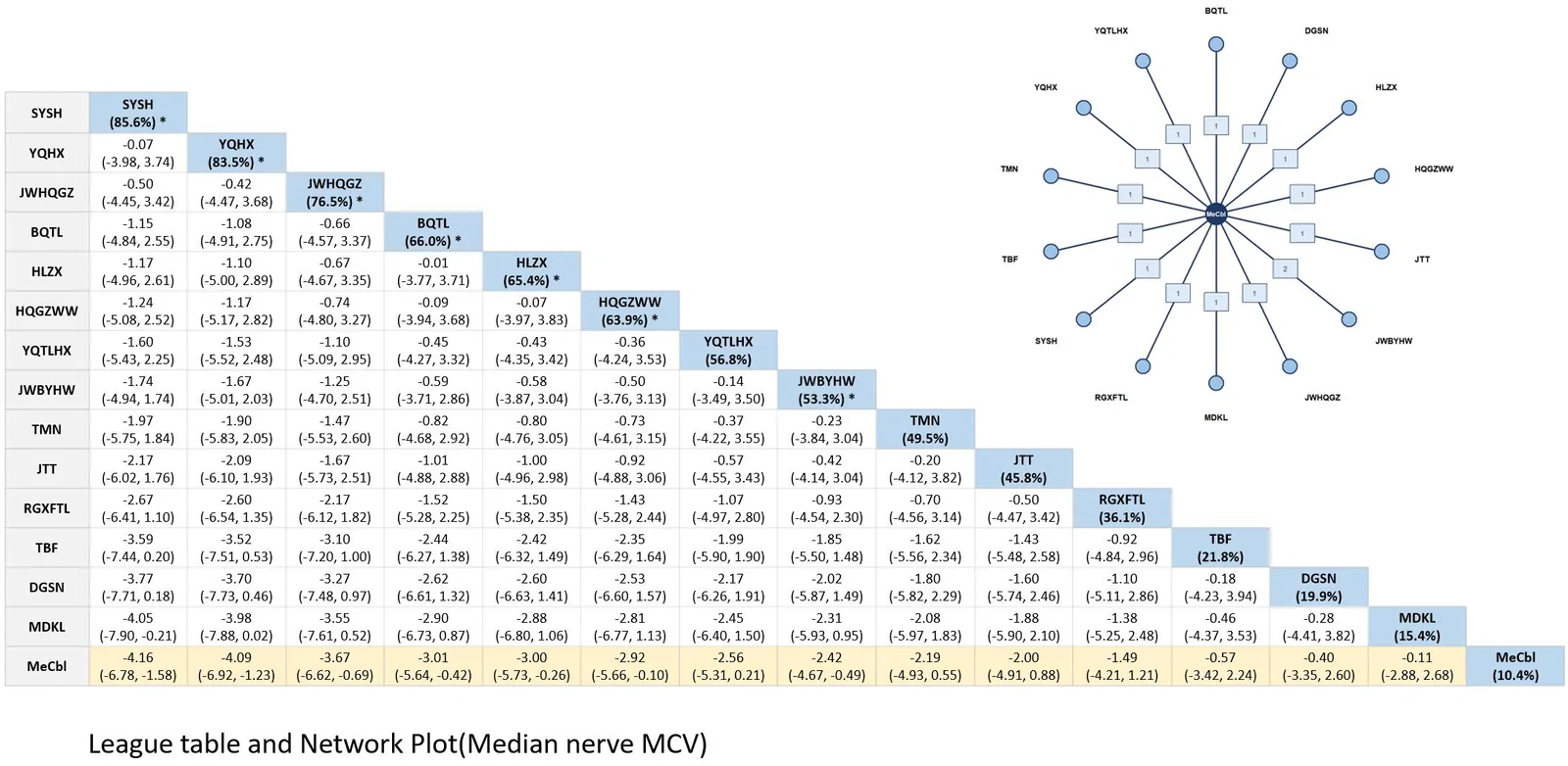
League table and network plot (median nerve MCV).

#### 3.2.3 Median nerve sensory conduction velocity

Fourteen studies (1,518 participants) reported median nerve SCV across 14 formulas. The 95% CrI excluded 0 for 3 formulas. By SUCRA, Xiaoma Zhitong Capsule ranked first (84.4%), Jiangtang Tongluo Tablet second (83.0%) and Yiqi Huashi Tongbi Formula third (75.4%), with mecobalamin ranked 13th (18.9%). The MD was 4.19 m/s (95% CrI 0.88 to 7.58) for Xiaoma Zhitong Capsule, 4.08 m/s (0.68 to 7.45) for Jiangtang Tongluo Tablet and 3.51 m/s (0.14 to 6.86) for Yiqi Huashi Tongbi Formula. The 95% CrI included 0 for the remaining 11 formulas (**Figure 5**).

**Figure 5:**
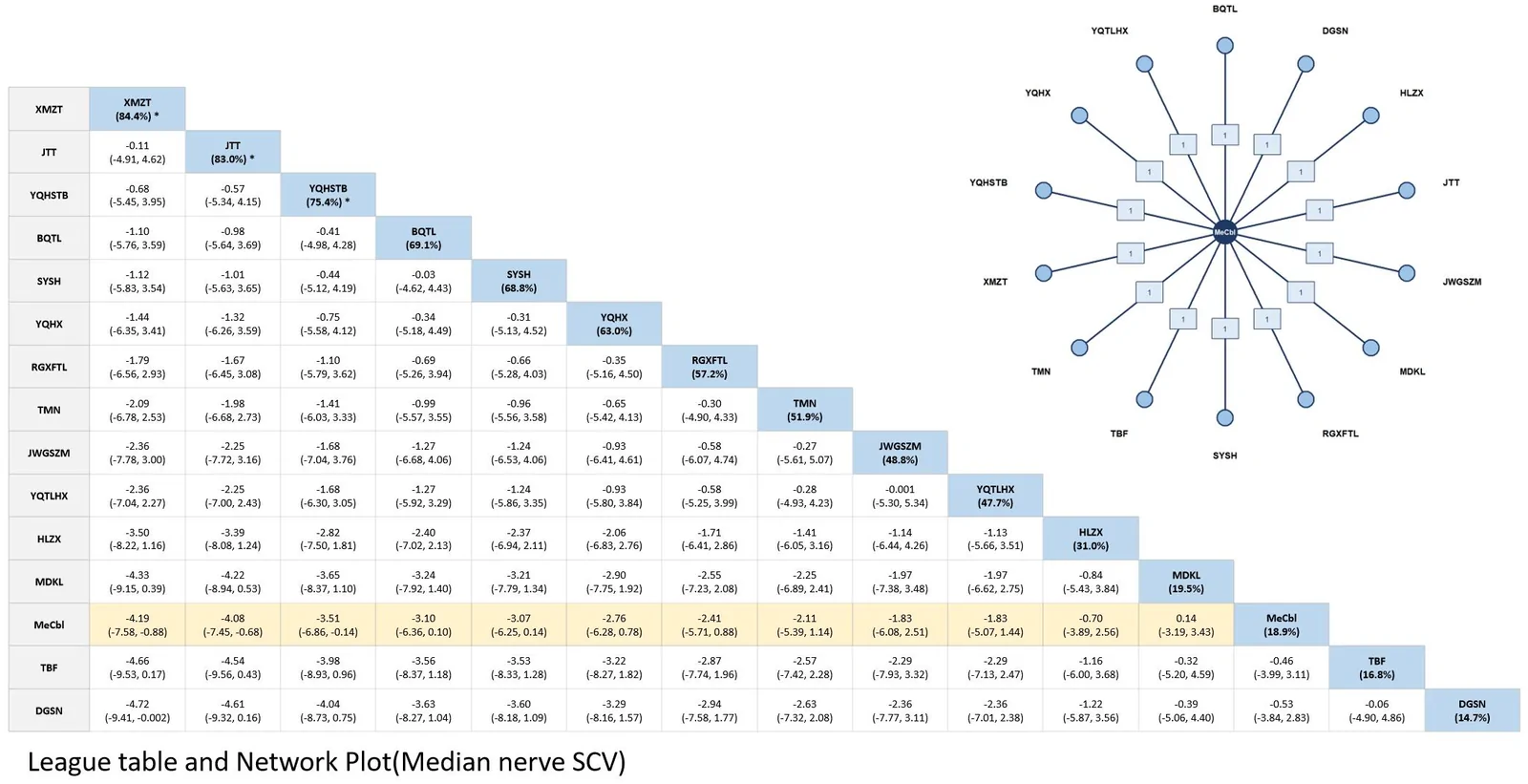
League table and network plot (median nerve SCV).

#### 3.2.4 Common peroneal nerve motor conduction velocity

Fourteen studies (1,585 participants) reported peroneal nerve MCV across 14 formulas. The 95% CrI excluded 0 for 5 formulas. By SUCRA, Modified Buyang Huanwu Decoction ranked first (79.2%), Modified Huangqi Guizhi Wuwu Decoction second (77.6%) and Danggui Sini Decoction third (73.9%), with mecobalamin ranked 14th (14.5%). The MD was 3.78 m/s (95% CrI 0.83 to 6.72) for Modified Buyang Huanwu Decoction and 3.72 m/s (0.64 to 6.81) for Modified Huangqi Guizhi Wuwu Decoction; the point estimate for Danggui Sini Decoction was 3.72 m/s, with a 95% CrI of -0.26 to 7.77 (**Figure 6**).

**Figure 6:**
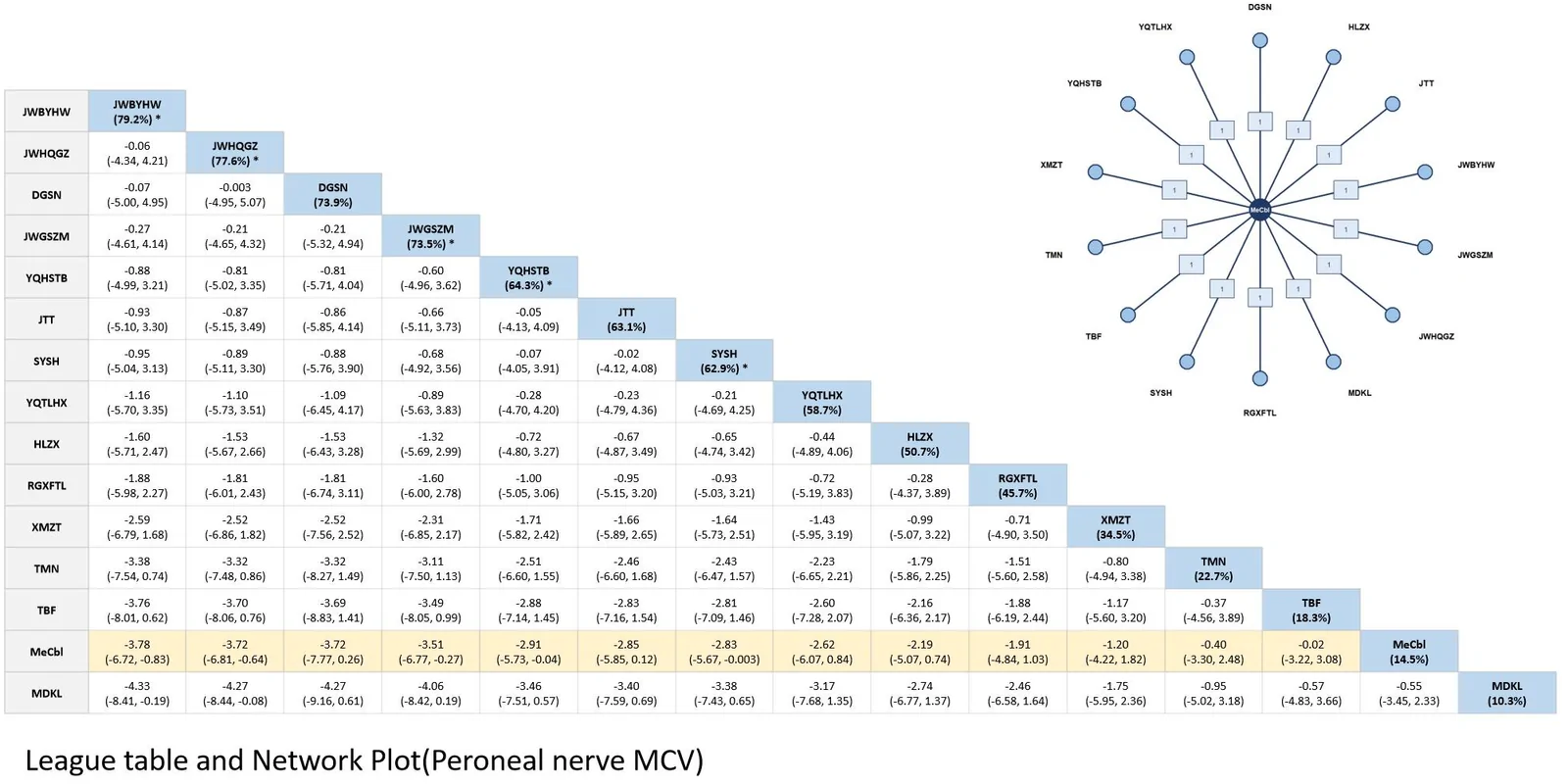
League table and network plot (peroneal nerve MCV).

#### 3.2.5 Common peroneal nerve sensory conduction velocity

Nine studies (879 participants) reported peroneal nerve SCV across 9 formulas. The 95% CrI excluded 0 for 8 formulas. By SUCRA, Jiangtang Tongluo Tablet ranked first (95.2%), Shengyang Sanhuo Decoction second (80.5%) and Buqi Tongluo Capsule third (61.7%), with mecobalamin last (1.6%). The MD was 4.08 m/s (95% CrI 2.38 to 5.78) for Jiangtang Tongluo Tablet, 3.09 m/s (1.66 to 4.47) for Shengyang Sanhuo Decoction and 2.44 m/s (0.98 to 3.89) for Buqi Tongluo Capsule. The 95% CrI included 0 for Xiaoma Zhitong Capsule (**Figure 7**).

**Figure 7:**
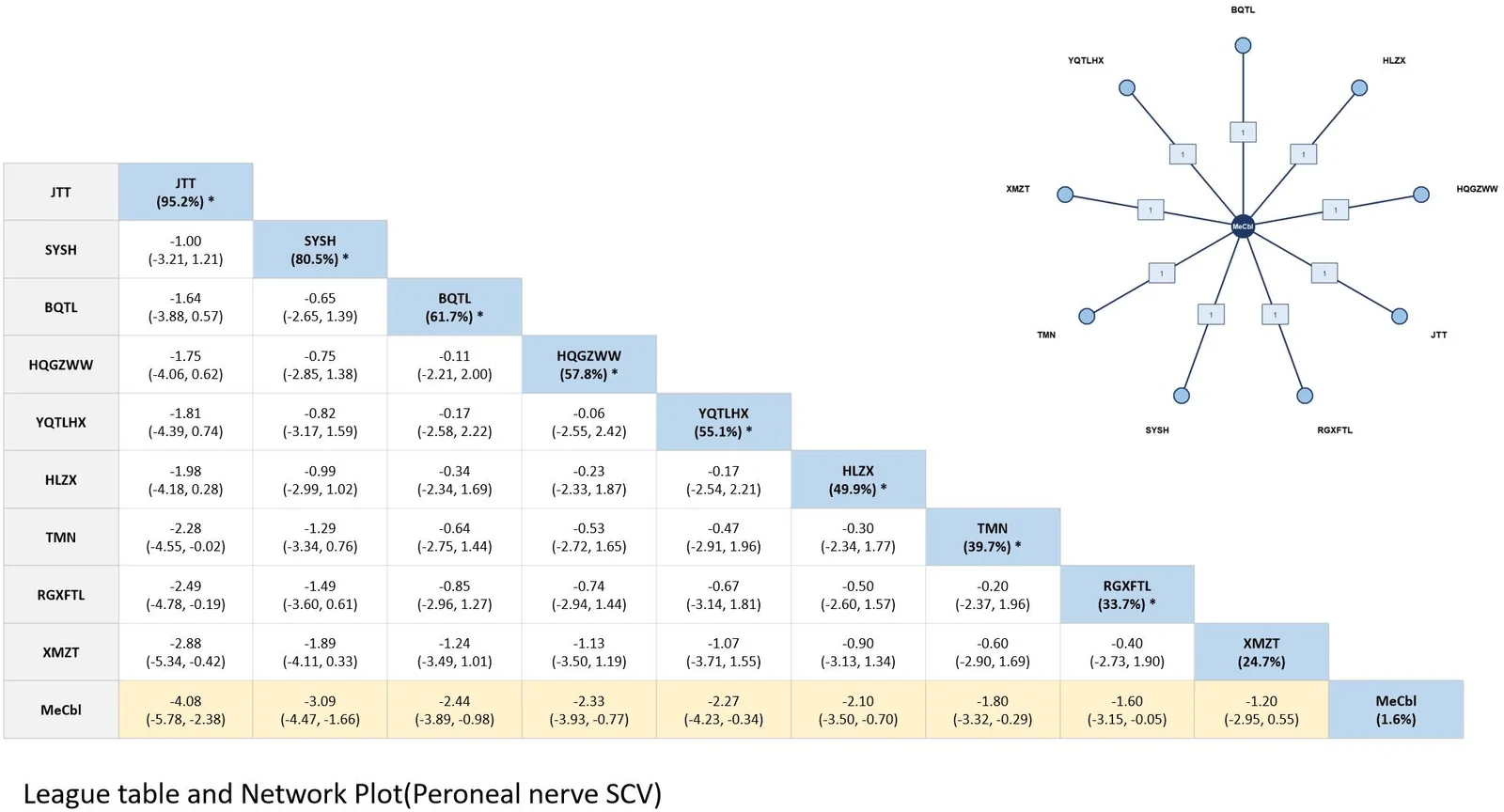
League table and network plot (peroneal nerve SCV).

#### 3.2.6 Heterogeneity and convergence diagnostics

The between-study variance at the network level was not identifiable for four of the five outcomes, the degrees of freedom for heterogeneity being zero. The Bayesian posterior tau-squared ranged from 0.241 to 1.351 under the half-normal prior and from 0.993 to 4.610 under the uniform prior. Across all models the maximum Gelman-Rubin potential scale reduction factor was 1.0013, the minimum effective sample size was 6,132, and the Monte Carlo standard error reached at most 1.28% of the posterior standard deviation. The mean residual deviance per data point ranged from 0.999 to 1.084 across models. Posterior heterogeneity and its identifiability are given in **Supplementary Tables 6a, b**, and convergence diagnostics and goodness of fit in **Supplementary Tables 7a, b**.

#### 3.2.7 Sensitivity analyses

The sensitivity analyses gave the following results. (i) In the leave-one-out analysis, each of the five outcomes had exactly one omission that changed the top-ranked formula, and in every case this occurred when the sole study informing the originally top-ranked node for that outcome was removed; under all other omissions the top-ranked formula for each outcome was unchanged (**Supplementary Table 9a**). (ii) In the node-redefinition analysis, only the Modified Buyang Huanwu Decoction node for median nerve MCV was formed by merging two studies; when this node was split into separate nodes and the model refitted, the top-ranked formula for that outcome was unchanged. All other nodes consisted of a single study and could not be subjected to a splitting analysis (**Supplementary Table 9b**). (iii) For studies in which the SD of the change score was derived from baseline and end-of-treatment values, varying the correlation coefficient from 0.5 to 0.9 left the top-ranked formula unchanged for all five outcomes; the point estimates were invariant to the correlation coefficient because each top-ranked node was informed by a single study and the mean difference contains no SD term. Confidence interval width narrowed as the correlation increased for four outcomes and widened for median nerve MCV; this sensitivity analysis was implemented with a frequentist network model (**Supplementary Table 8**).

### 3.3 Pairwise meta-analysis

Pooled pairwise results for each outcome were as follows. TCSS: MD -2.14 (95% CI -2.66 to -1.61), I-squared 90.5%, prediction interval -3.86 to -0.41. Median nerve MCV: MD 2.39 m/s (1.66 to 3.11), I-squared 82.6%, prediction interval -0.17 to 4.94. Median nerve SCV: MD 2.06 m/s (1.14 to 2.98), I-squared 86.0%, prediction interval -1.21 to 5.32. Peroneal nerve MCV: MD 2.13 m/s (1.28 to 2.98), I-squared 82.9%, prediction interval -0.76 to 5.02. Peroneal nerve SCV: MD 2.33 m/s (1.71 to 2.94), I-squared 66.8%, prediction interval 0.79 to 3.86. Pairwise forest plots for all outcomes are shown in **Figure 8**.

**Figure 8:**
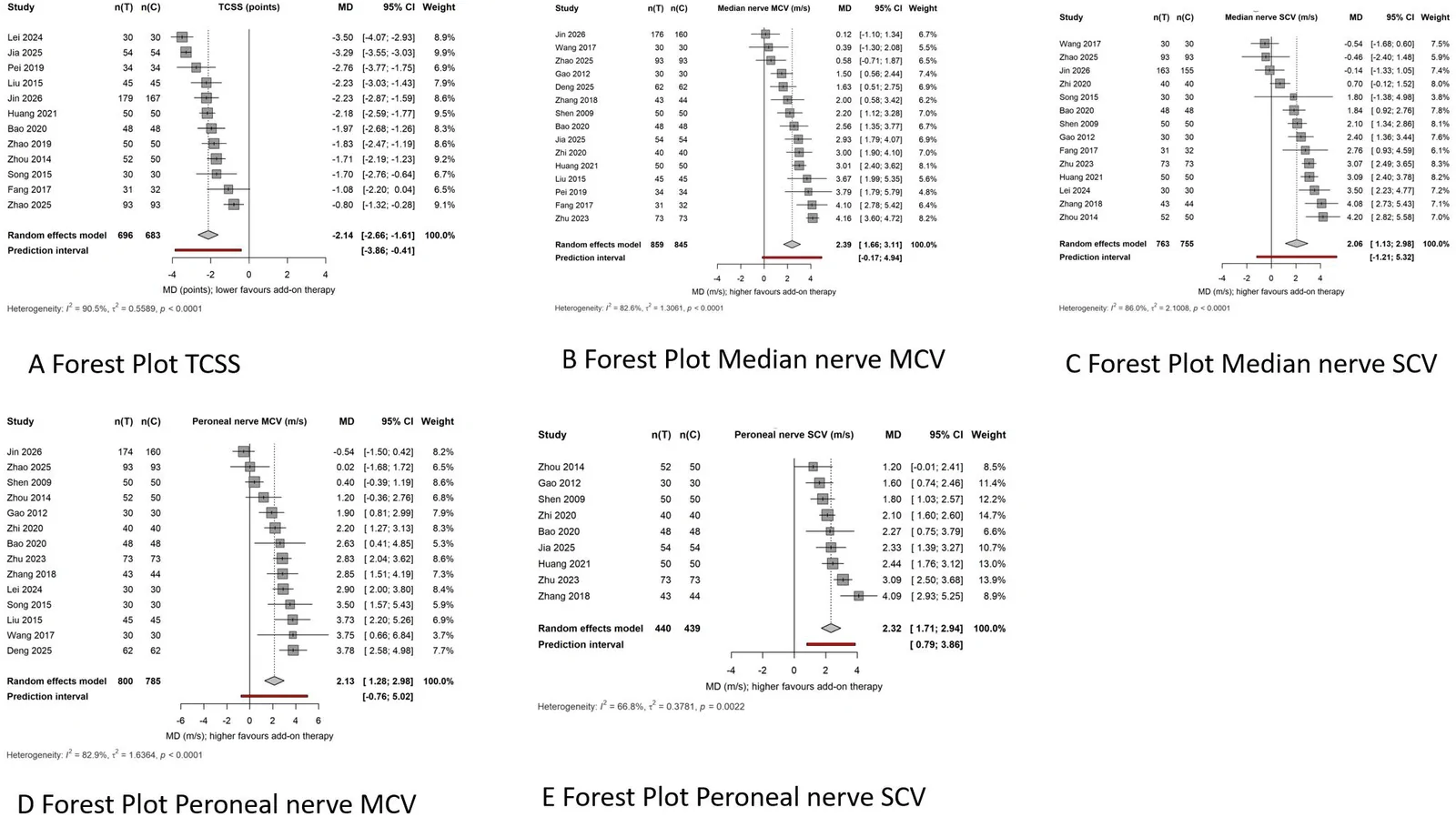
Comparison of therapeutic effects between add-on therapy and control group (Forest plot). **(A–E)** show the comparison of mean differences (MD) between the add-on therapy group and the control group for the following indicators: **(A)** TCSS score; **(B)** Median nerve motor conduction velocity (MCV); **(C)** Median nerve sensory conduction velocity (SCV); **(D)** Peroneal nerve motor conduction velocity (MCV); **(E)** Peroneal nerve sensory conduction velocity (SCV).

Prediction intervals crossed the line of no effect for three outcomes: median nerve MCV, median nerve SCV and peroneal nerve MCV. The pairwise REML estimate of tau-squared ranged from 0.378 to 2.101 (**Supplementary Table 5**).

### 3.4 Risk of bias and certainty of evidence

Risk of bias was assessed separately for subjective and objective outcomes (**Figure 9**). All trials raised some concerns in the domains of the randomisation process and selection of the reported result, chiefly because allocation concealment, trial registration and study protocols were inadequately reported. The outcome measurement domain differed markedly between the two outcome types: most trials reporting TCSS were open-label and did not report blinding of outcome assessors, and this domain was mostly rated at high risk, whereas trials reporting nerve conduction velocity used instrument-based outcomes and this domain was generally rated as raising some concerns. Study-by-study and domain-by-domain judgements are shown in **Figure 9**.

**Figure 9:**
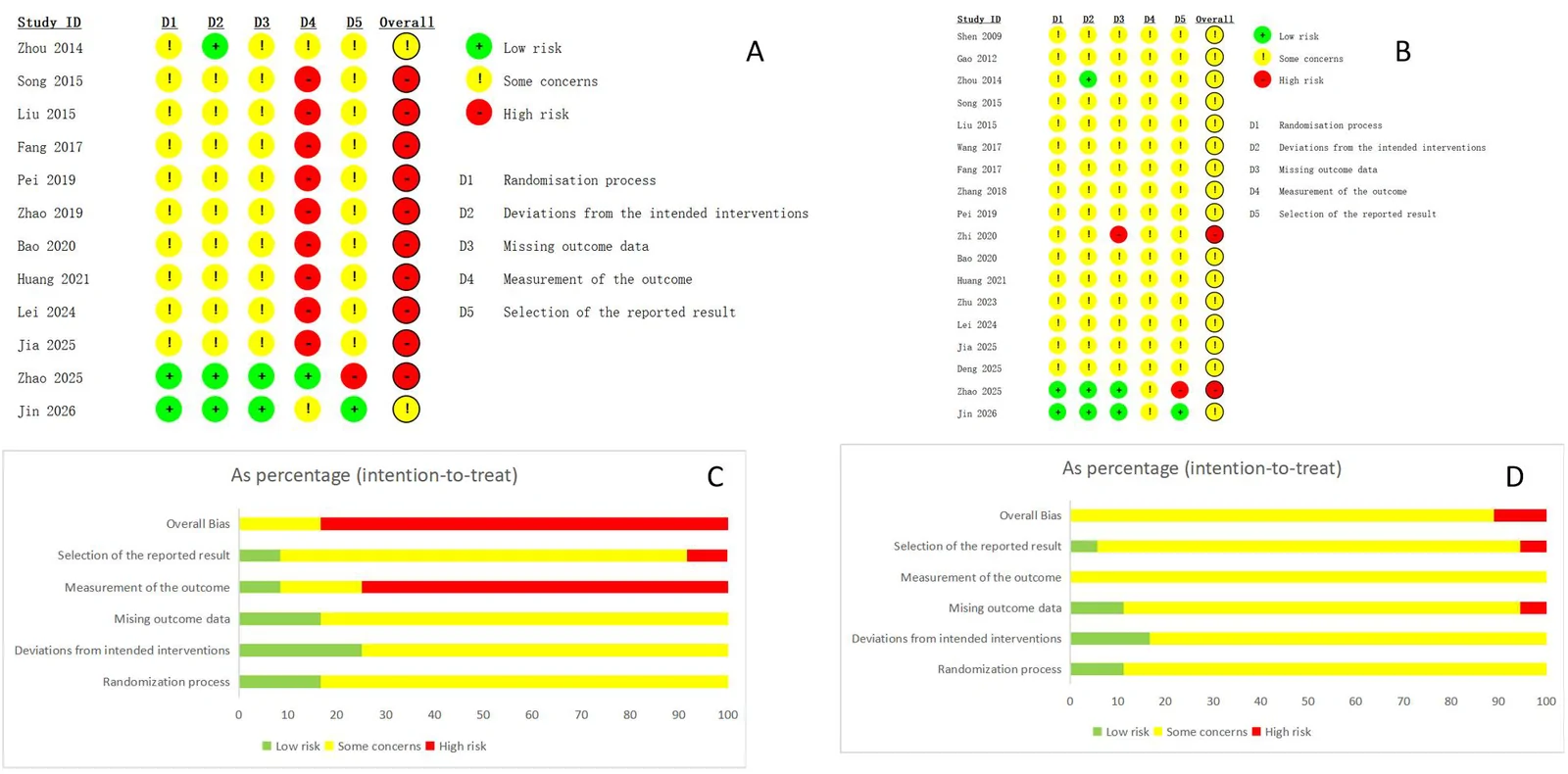
Risk of bias in the included trials, assessed with the Cochrane risk-of-bias tool for randomized trials (RoB 2). **(A, C)** Subjective outcome (Toronto Clinical Scoring System; 12trials). **(B, D)** Objective outcomes (nerve conduction velocity; 18 trials). **(A, B)** Traffic-light plots showing domain-level and overall judgements for each trial. **(C, D)** Summary plots showing the distribution of judgements across trials as percentanges. D1, randomisation process; D2, devations from the intended interventions; D3, missing outcome data; D4, measurement of the outcome; D5, selection of the reported result.

The certainty of evidence for every network estimate was assessed with CINeMA (version 2.0.0) and was very low throughout. Downgrading was driven mainly by within-study bias, indirectness and heterogeneity, with imprecision providing an additional reason for downgrading some estimates. Because the networks contain no closed loops, incoherence could not be evaluated empirically. Comparison-by-comparison judgements are given in **Supplementary Table 11**.

### 3.5 Small-study effects and publication bias

Category-level funnel plots are shown in **Figure 2**. For each outcome the points were distributed on either side of the random-effects pooled estimate, with no marked asymmetry. P values for Egger’s regression, the Begg-Mazumdar rank correlation and the Thompson-Sharp test ranged from 0.065 to 0.977, and none reached 0.05; Egger’s test gave P values of 0.079 for TCSS and 0.065 for median nerve MCV, close to the conventional threshold (**Supplementary Table 10**). Peroneal nerve SCV included only 9 trials, fewer than the 10 usually required for tests of funnel plot asymmetry. Statistical power was therefore limited, and the absence of a significant result should not be taken as evidence that publication bias is absent.

### 3.6 Adverse events

Of the 19 studies, 4 reported the number of adverse events by arm, 1 reported event counts without indicating the arm, 6 stated only that no obvious adverse reactions were observed without providing event categories or counts, and 8 made no mention of adverse-event monitoring. Three studies pre-specified laboratory safety assessments, but none reported the corresponding numerical values. None of the 19 studies stated whether adverse events were collected by active monitoring or by passive reporting, and none reported hypoglycaemia or formula-drug interactions.

The events reported were confined to mild gastrointestinal reactions and raised liver enzymes. Jia 2025 (36) reported 7 events (12.96%) in the intervention arm and 5 (9.26%) in the control arm, a difference that was not statistically significant (chi-square = 0.375, P > 0.05). Jin 2026 (39) reported any adverse event in 94/200 versus 82/197, serious adverse events in 10 versus 9 and adverse drug reactions in 12 versus 3, with hepatobiliary abnormalities the most frequent category. Zhou 2014 (23) reported 2 cases of nausea and retching in the intervention arm, both of which resolved after discontinuation. No study reported a fatal event related to the intervention.

## 4 Discussion

### 4.1 Principal findings and clinical positioning

By SUCRA, Yiqi Huashi Tongbi Formula ranked first for TCSS (SUCRA 90.3%; MD -3.49, 95% CrI -5.17 to -1.81), Shengyang Sanhuo Decoction for median nerve MCV (SUCRA 85.6%; MD 4.16, 95% CrI 1.58 to 6.78), Xiaoma Zhitong Capsule for median nerve SCV (SUCRA 84.4%; MD 4.19, 95% CrI 0.88 to 7.58), Modified Buyang Huanwu Decoction for peroneal nerve MCV (SUCRA 79.2%; MD 3.78, 95% CrI 0.83 to 6.72) and Jiangtang Tongluo Tablet for peroneal nerve SCV (SUCRA 95.2%; MD 4.08, 95% CrI 2.38 to 5.78).

The clinical meaning of these effect sizes calls for cautious interpretation. No minimal clinically important difference (MCID) has been established for the TCSS. An improvement in TCSS after the addition of traditional Chinese medicine is therefore not equivalent to a threshold of benefit perceptible to patients, which would have to be established from direct patient-reported outcome evidence.

For nerve conduction velocity, the work of Dyck and colleagues offers a reference point, indicating that a clinically meaningful change in common peroneal nerve motor conduction velocity (MCV) is approximately 2.2 m/s (40). Improvements in the nerve conduction measures in the present review were of the order of 2 to 4 m/s, and for some formulas exceeded this reference value. That reference, however, applies to common peroneal nerve motor conduction velocity, and its suitability as a threshold for interpreting treatment effects on nerve conduction velocity has not been independently validated. Nerve conduction velocity is moreover a surrogate endpoint, and its actual bearing on patients’ sensory and motor function remains unclear.

Although these effect sizes meet or exceed existing reference standards numerically, their translational significance remains uncertain, and they should not be read directly as establishing the clinical superiority of any formula. Future work should seek to confirm the present findings in well-designed head-to-head randomised controlled trials that incorporate more clinically relevant measures such as patient-reported outcomes.

### 4.2 Strengths and limitations

The methodological strengths of this review are as follows.

- In terms of the evidence base, inclusion was restricted strictly to randomised controlled trials, which preserves the benefit of randomisation in the primary trials and minimises confounding bias.
- In terms of bias assessment, RoB 2 was applied on an outcome-specific basis, with the outcome measurement domain judged separately for the subjective outcome (TCSS) and the objective outcomes (nerve conduction velocity). Certainty of evidence was rated with the CINeMA framework for every network estimate, covering each direct and indirect comparison.
- Transitivity was formally evaluated. The distributions of effect modifiers were compared across treatment nodes, and their between-node ranges, coefficients of variation and missing-data rates were reported.

#### 4.2.1 Limitations

The results of this review must be read within the following limitations.

First, structural constraints of the evidence network. All five outcome networks are star-shaped: each blood-activating, collateral-unblocking formula forms a direct comparison only with mecobalamin, and every comparison between formulas rests on indirect evidence. This structure imposes several inherent statistical limitations. The number of closed loops is zero, so inconsistency cannot be tested statistically by node-splitting or by any other method. The degrees of freedom for heterogeneity are zero for four outcomes, so the network-level heterogeneity parameter is not identifiable; in a Bayesian framework this means that the posterior distribution of tau-squared is driven principally by the prior specification rather than informed by the data, and the heterogeneity estimate therefore carries no information from the data. The interpretability of comparison-adjusted funnel plots is limited, and small-study effects can be examined only at the category level, not at the level of individual formulas. Furthermore, because all nodes but one are informed by a single trial, omitting a study in the leave-one-out analysis simultaneously removes the node to which it belongs; what can then be tested is only the relative ranking of the remaining nodes, not the robustness of the effect estimate for the removed node itself. The ranking metrics reported here therefore reflect probabilistic ordering rather than the precision of the effect estimates, and should be regarded as exploratory.

Second, incomplete justification of the transitivity assumption. Within the framework of traditional Chinese medicine theory, the patterns targeted by individual blood-activating, collateral-unblocking formulas differ to some degree, and the pathogenesis in a given patient is often not singular but may involve several coexisting pathological states and their causal relationships; the assumption that patients are exchangeable across formula nodes therefore does not hold fully. The trials also differed in treatment duration (4 to 24 weeks), background treatment regimen and the conditions under which nerve conduction was measured, all of which may influence the effect estimates. Among the key effect modifiers, missing-data rates were high for duration of diabetes, duration of DPN, glycated haemoglobin and fasting plasma glucose, so transitivity could not be systematically evaluated on these dimensions.

Third, methodological quality and completeness of reporting in the primary trials. Apart from two internationally registered multicentre double-blind placebo-controlled trials, all were single-centre trials conducted in China, and most did not blind participants or investigators. Among the trials reporting TCSS, most did not state whether outcome assessors were blinded. As regards reporting of the interventions, no trial provided quality control information such as batch numbers of decoction pieces, content determination or chromatographic fingerprinting. As regards safety reporting, 8 trials made no mention whatsoever of adverse-event monitoring, and none of the 19 trials reported numerical laboratory safety data. Given these shortcomings, the available evidence is insufficient to support a meaningful assessment of benefit and risk.

Fourth, the limited scope of the conclusions. Effects in this review were evaluated only for two surrogate measures, TCSS and nerve conduction velocity, and do not extend to endpoints of greater clinical importance to patients, such as pain relief, physical function, quality of life, foot ulceration, amputation or long-term progression of neuropathy.

Fifth, limitations at the level of the model and of the certainty rating. Most formula nodes are informed by a single trial and the network structure is highly sparse. Under these conditions the model is close to saturated: the effective number of parameters (pD) is comparable to the number of data points, the fit and penalty terms both approach the number of data points, and the DIC difference between the common-effect and random-effects models amounts to only 0.01 to 1.36. Residual deviance and DIC are therefore unsuitable for judging the adequacy of model fit and cannot serve as a basis for choosing between the two model types. The degrees of freedom for heterogeneity are zero for four of the five outcomes, and in these four outcomes the width of the network estimates reflects the heterogeneity specified by the prior rather than heterogeneity estimated from the data. This review used TCSS as the primary outcome together with four nerve conduction outcomes, and no correction was made for multiple comparisons across outcomes. All network estimates were rated very low in certainty by CINeMA, and among the six domains only imprecision varied between comparisons, so the rating does not differentiate the relative credibility of individual comparisons. Finally, the tests for small-study effects had limited power, and a non-significant result cannot be taken as evidence that publication bias is absent.

## 5 Conclusion

This Bayesian network meta-analysis compared the relative effects of blood-activating, collateral-unblocking traditional Chinese medicine formulas given as add-on therapy to mecobalamin. Most formulas were associated with improvement in TCSS and in nerve conduction velocity, but the top-ranked formula differed between outcomes, every comparison between formulas rested on indirect evidence, and the certainty of all network estimates was very low. These findings are exploratory and may serve to indicate directions for further research, but they are not sufficient to support the selection of a particular formula for an individual patient, nor do they indicate that any formula has established clinical superiority. Head-to-head comparisons between formulas are still lacking; adequately powered, prospectively registered, blinded multicentre studies applying uniform outcome assessment and systematic collection of safety data remain necessary.

## Data Availability

The original contributions presented in the study are included in the article/**Supplementary Material**. Further inquiries can be directed to the corresponding author.
