# Supplementary material for: Blood-activating and collateral-unblocking traditional Chinese medicine formulas combined with mecobalamin for peripheral neuropathy in type 2 diabetes: a systematic review and Bayesian network meta-analysis
Source: Front Endocrinol (Lausanne). 2026 Sep 16;17:1888494. doi: 10.3389/fendo.2026.1888494 (PMC13623713; doi:10.3389/fendo.2026.1888494)
Supplement: Supplementary file 1 [file Supplementaryfile1.docx]

# Supplementary Tables

## Table S1. PRISMA 2020 checklist

| **Section and Topic** | **Item #** | **Checklist item** | **Location where item is reported** |
| --- | --- | --- | --- |
| Title | 1 | Identify the report as a systematic review. | Title |
| Abstract | 2 | See the PRISMA 2020 for Abstracts checklist. | Abstract |
| Rationale | 3 | Describe the rationale for the review in the context of existing knowledge. | Section 1 (Introduction) |
| Objectives | 4 | Provide an explicit statement of the objective(s) or question(s) the review addresses. | Section 1 (Introduction); Abstract, Background |
| Eligibility criteria | 5 | Specify the inclusion and exclusion criteria for the review and how studies were grouped for the syntheses. | Sections 2.2, 2.3, 2.4 and 2.6 |
| Information sources | 6 | Specify all databases, registers, websites, organisations, reference lists and other sources searched or consulted to identify studies. Specify the date when each source was last searched or consulted. | Section 2.5; Supplementary Table S2 |
| Search strategy | 7 | Present the full search strategies for all databases, registers and websites, including any filters and limits used. | Section 2.5; Supplementary Table S2 |
| Selection process | 8 | Specify the methods used to decide whether a study met the inclusion criteria of the review, including how many reviewers screened each record and each report retrieved, whether they worked independently, and if applicable, details of automation tools used in the process. | Section 2.6; Figure 1 |
| Data collection process | 9 | Specify the methods used to collect data from reports, including how many reviewers collected data from each report, whether they worked independently, any processes for obtaining or confirming data from study investigators, and if applicable, details of automation tools used in the process. | Section 2.6 |
| Data items | 10a | List and define all outcomes for which data were sought. Specify whether all results that were compatible with each outcome domain in each study were sought (e.g. for all measures, time points, analyses), and if not, the methods used to decide which results to collect. | Section 2.4 |
|  | 10b | List and define all other variables for which data were sought (e.g. participant and intervention characteristics, funding sources). Describe any assumptions made about any missing or unclear information. | Section 2.6; Table 1; Tables 2a and 2b; Supplementary Tables S3a and S3b |
| Study risk of bias assessment | 11 | Specify the methods used to assess risk of bias in the included studies, including details of the tool(s) used, how many reviewers assessed each study and whether they worked independently, and if applicable, details of automation tools used in the process. | Section 2.7 |
| Effect measures | 12 | Specify for each outcome the effect measure(s) (e.g. risk ratio, mean difference) used in the synthesis or presentation of results. | Sections 2.8.1 and 2.8.2 |
| Synthesis methods | 13a | Describe the processes used to decide which studies were eligible for each synthesis (e.g. tabulating the study intervention characteristics and comparing against the planned groups for each synthesis (item #5)). | Sections 2.4 and 2.8.2 |
|  | 13b | Describe any methods required to prepare the data for presentation or synthesis, such as handling of missing summary statistics, or data conversions. | Section 2.6 (conversion of change-score standard deviations); Supplementary Table S8 |
|  | 13c | Describe any methods used to tabulate or visually display results of individual studies and syntheses. | Sections 2.8.2 and 2.8.4; Figures 3 to 9 |
|  | 13d | Describe any methods used to synthesize results and provide a rationale for the choice(s). If meta-analysis was performed, describe the model(s), method(s) to identify the presence and extent of statistical heterogeneity, and software package(s) used. | Sections 2.8.1, 2.8.2 and 2.8.3 |
|  | 13e | Describe any methods used to explore possible causes of heterogeneity among study results (e.g. subgroup analysis, meta-regression). | Subgroup analysis and meta-regression were not undertaken because most treatment nodes were informed by a single trial; the consequences are discussed in Section 4.2. |
|  | 13f | Describe any sensitivity analyses conducted to assess robustness of the synthesized results. | Section 2.8.5 |
| Reporting bias assessment | 14 | Describe any methods used to assess risk of bias due to missing results in a synthesis (arising from reporting biases). | Section 2.8.5 |
| Certainty assessment | 15 | Describe any methods used to assess certainty (or confidence) in the body of evidence for an outcome. | Section 2.7 |
| Study selection | 16a | Describe the results of the search and selection process, from the number of records identified in the search to the number of studies included in the review, ideally using a flow diagram. | Section 3.1; Figure 1 |
|  | 16b | Cite studies that might appear to meet the inclusion criteria, but which were excluded, and explain why they were excluded. | Figure 1 |
| Study characteristics | 17 | Cite each included study and present its characteristics. | Section 3.1; Table 1; Tables 2a and 2b; Supplementary Tables S3a and S3b |
| Risk of bias in studies | 18 | Present assessments of risk of bias for each included study. | Section 3.4; Figure 2 |
| Results of individual studies | 19 | For all outcomes, present, for each study: (a) summary statistics for each group (where appropriate) and (b) an effect estimate and its precision (e.g. confidence/credible interval), ideally using structured tables or plots. | Figure 8; Supplementary Tables S3a and S3b |
| Results of syntheses | 20a | For each synthesis, briefly summarise the characteristics and risk of bias among contributing studies. | Sections 3.1 and 3.4; Supplementary Table S4 |
|  | 20b | Present results of all statistical syntheses conducted. If meta-analysis was done, present for each the summary estimate and its precision (e.g. confidence/credible interval) and measures of statistical heterogeneity. If comparing groups, describe the direction of the effect. | Sections 3.2 and 3.3; Figures 3 to 8; Supplementary Table S5 |
|  | 20c | Present results of all investigations of possible causes of heterogeneity among study results. | Section 3.2.6 and Supplementary Tables S6a, S6b, S7a and S7b. |
|  | 20d | Present results of all sensitivity analyses conducted to assess the robustness of the synthesized results. | Section 3.2.7; Supplementary Tables S8, S9a and S9b |
| Reporting biases | 21 | Present assessments of risk of bias due to missing results (arising from reporting biases) for each synthesis assessed. | Section 3.5; Figure 9; Supplementary Table S10 |
| Certainty of evidence | 22 | Present assessments of certainty (or confidence) in the body of evidence for each outcome assessed. | Section 3.4; Supplementary Table S11 |
| Discussion | 23a | Provide a general interpretation of the results in the context of other evidence. | Section 4.1 |
|  | 23b | Discuss any limitations of the evidence included in the review. | Section 4.2 (Limitations) |
|  | 23c | Discuss any limitations of the review processes used. | Section 4.2 (Limitations) |
|  | 23d | Discuss implications of the results for practice, policy, and future research. | Sections 4.1 and 5 |
| Registration and protocol | 24a | Provide registration information for the review, including register name and registration number, or state that the review was not registered. | Section 2.1 |
|  | 24b | Indicate where the review protocol can be accessed, or state that a protocol was not prepared. | Section 2.1 |
|  | 24c | Describe and explain any amendments to information provided at registration or in the protocol. | Section 2.1 |
| Support | 25 | Describe sources of financial or non-financial support for the review, and the role of the funders or sponsors in the review. | Funding statement |
| Competing interests | 26 | Declare any competing interests of review authors. | Conflict of interest statement |
| Availability of data, code and other materials | 27 | Report which of the following are publicly available and where they can be found: template data collection forms; data extracted from included studies; data used for all analyses; analytic code; any other materials used in the review. | Data availability statement; Section 2.5 and Supplementary Table S2 (search strategies) |

## Table S2. Search strategies

### MEDLINE (PubMed)

#1	"Diabetic Neuropathies"[Mesh]

#2	"Diabetic Foot"[Mesh]

#3	(diabet*[tiab] AND (neuropath*[tiab] OR polyneuropath*[tiab] OR neuritis[tiab]

OR "nerve conduction"[tiab] OR neuralgia[tiab]))

#4	DPN[tiab] OR DSPN[tiab]

#5	#1 OR #2 OR #3 OR #4

#6	"Medicine, Chinese Traditional"[Mesh]

#7	"Drugs, Chinese Herbal"[Mesh]

#8	"Medicine, East Asian Traditional"[Mesh]

#9	"Phytotherapy"[Mesh]

#10 ("Chinese herbal"[tiab] OR "Chinese medicine"[tiab] OR "traditional Chinese"[tiab]

OR TCM[tiab] OR decoction*[tiab] OR granule*[tiab] OR "Chinese patent medicine"[tiab]

OR "herbal formula"[tiab] OR "herbal preparation"[tiab])

#11 ("activating blood"[tiab] OR "blood-activating"[tiab]

OR "promoting blood circulation"[tiab] OR "removing blood stasis"[tiab]

OR "resolving stasis"[tiab] OR "dredging collateral"[tiab]

OR "unblocking collateral"[tiab] OR huoxue[tiab] OR tongluo[tiab]

OR huayu[tiab] OR "blood stasis"[tiab])

#12 #6 OR #7 OR #8 OR #9 OR #10 OR #11

#13 ("randomized controlled trial"[pt] OR "controlled clinical trial"[pt]

OR randomized[tiab] OR randomised[tiab] OR placebo[tiab] OR randomly[tiab]

OR trial[ti] OR "clinical trials as topic"[Mesh:noexp])

NOT ("animals"[Mesh] NOT "humans"[Mesh])

#14 #5 AND #12 AND #13

### Embase

#1	'diabetic neuropathy'/exp

#2	(diabet* NEAR/3 (neuropath* OR polyneuropath* OR neuritis)):ti,ab,kw

#3	#1 OR #2

#4	'Chinese medicine'/exp OR 'Chinese drug'/exp OR 'herbal medicine'/exp

#5	(('chinese' NEAR/2 (herb* OR medicin* OR drug*)) OR decoction* OR granule*

OR 'chinese patent medicine' OR huoxue OR tongluo OR 'blood stasis'

OR 'activating blood' OR 'promoting blood circulation'):ti,ab,kw

#6	#4 OR #5

#7	'randomized controlled trial'/exp OR 'controlled clinical trial'/exp

OR random*:ti,ab OR placebo:ti,ab OR 'double blind':ti,ab

#8	#3 AND #6 AND #7

#9	#8 NOT ([animals]/lim NOT [humans]/lim)

### Cochrane Central Register of Controlled Trials (CENTRAL)

#1 MeSH descriptor: [Diabetic Neuropathies] explode all trees

#2 ((diabet* NEAR/3 (neuropath* or polyneuropath* or neuritis)) or DPN or DSPN):ti,ab,kw

#3 #1 OR #2

#4 MeSH descriptor: [Medicine, Chinese Traditional] explode all trees

#5 MeSH descriptor: [Drugs, Chinese Herbal] explode all trees

#6 MeSH descriptor: [Phytotherapy] explode all trees

#7 ((chinese NEAR/2 (herb* or medicin* or drug*)) or decoction* or granule* or "Chinese patent medicine" or "herbal formula" or "herbal preparation" or huoxue or tongluo or "blood stasis"):ti,ab,kw

#8 #4 OR #5 OR #6 OR #7

#9 #3 AND #8, in Trials

### Web of Science Core Collection

#1	TS=("diabetic neuropath*" OR "diabetic peripheral neuropath*" OR DPN OR DSPN)

#2	TS=(diabet* NEAR/3 (neuropath* OR polyneuropath* OR neuritis))

#3	#1 OR #2

#4	TS=("Chinese herb*" OR "Chinese herbal" OR "traditional Chinese medicine" OR TCM

OR "Chinese patent medicine" OR "proprietary Chinese medicine"

OR "integrated traditional Chinese and Western medicine"

OR "integrated Chinese and Western" OR "herbal medicine" OR "herbal remedy"

OR decoction* OR granule* OR "oral liquid" OR "medicinal drink" OR pill*

OR pellet* OR powder* OR "blood stasis" OR "activating blood"

OR "blood-activating" OR "promoting blood circulation"

OR "blood circulation promoting" OR "unblocking collaterals")

#5	TS=(random* OR RCT OR "controlled trial" OR placebo OR blind*)

#6	#3 AND #4 AND #5

### CNKI (China National Knowledge Infrastructure)

Field code SU = subject; + denotes OR.

S1	SU=('糖尿病周围神经病变'+'糖尿病性周围神经病变'+'糖尿病末梢神经炎'

+'糖尿病神经病变'+'DPN'+'消渴痹症')

[Subject = ('diabetic peripheral neuropathy', two Chinese variants

OR 'diabetic distal neuritis' OR 'diabetic neuropathy' OR 'DPN'

OR 'Xiaoke Bi syndrome')]

S2	SU=('中医'+'中医药'+'中西医结合'+'中药'+'中草药'+'中成药'+'复方'+'汤剂')

[Subject = ('traditional Chinese medicine' OR 'Chinese medicine and pharmacy'

OR 'integrated Chinese and Western medicine' OR 'Chinese herbal medicine'

OR 'Chinese medicinal herbs' OR 'Chinese patent medicine'

OR 'compound formula' OR 'decoction')]

S3	SU=('活血'+'通络'+'化瘀'+'祛瘀')

[Subject = ('activating blood' OR 'unblocking collaterals'

OR 'resolving stasis' OR 'dispelling stasis')]

S4	SU=('甲钴胺'+'弥可保'+'甲基钴胺素')

[Subject = ('mecobalamin' OR 'Methycobal' OR 'methylcobalamin')]

S5	S1 AND S2

S6	S5 AND S3

S7	S6 AND S4

### Wanfang Data

Field code 主题 = subject.

S1	主题:("糖尿病周围神经病变" or "糖尿病性周围神经病变" or "糖尿病末梢神经炎"

or "糖尿病神经病变" or "DPN" or "消渴痹症")

[Subject: same term set as CNKI line S1]

S2	主题:("中医" or "中医药" or "中西医结合" or "中药" or "中草药"

or "中成药" or "复方" or "汤剂")

[Subject: same term set as CNKI line S2]

S3	主题:("活血" or "通络" or "化瘀" or "祛瘀")

[Subject: same term set as CNKI line S3]

S4	主题:("甲钴胺" or "弥可保" or "甲基钴胺素")

[Subject: same term set as CNKI line S4]

S5	S1 AND S2

S6	S5 AND S3

S7	S6 AND S4

### VIP (CQVIP Chinese Science and Technology Journal Database)

Field code M = title, keywords and abstract (篇关摘)

S1	M=(糖尿病周围神经病变 + 糖尿病性周围神经病变 + 糖尿病末梢神经炎

+ 糖尿病神经病变 + DPN + 消渴痹症)

[Title, keywords or abstract = ('diabetic peripheral neuropathy', two Chinese

variants OR 'diabetic distal neuritis' OR 'diabetic neuropathy' OR 'DPN'

OR 'Xiaoke Bi syndrome')]

S2	M=(中医 + 中医药 + 中西医结合 + 中药 + 中草药 + 中成药 + 复方 + 汤剂)

[Title, keywords or abstract = ('traditional Chinese medicine'

OR 'Chinese medicine and pharmacy' OR 'integrated Chinese and Western

medicine' OR 'Chinese herbal medicine' OR 'Chinese medicinal herbs'

OR 'Chinese patent medicine' OR 'compound formula' OR 'decoction')]

S3	M=(活血 + 通络 + 化瘀 + 祛瘀)

[Title, keywords or abstract = ('activating blood' OR 'unblocking collaterals'

OR 'resolving stasis' OR 'dispelling stasis')]

S4	M=(甲钴胺 + 弥可保 + 甲基钴胺素)

[Title, keywords or abstract = ('mecobalamin' OR 'Methycobal'

OR 'methylcobalamin')]

S5	S1 AND S2

S6	S5 AND S3

S7	S6 AND S4

## Table S3a. Demographic and metabolic characteristics of the included trials

| **Study** | **Node** | **No. randomised (T/C)** | **Baseline HbA1c, % (T/C)** | **Baseline FPG, mmol/L (T/C)** | **Age, years (T/C)** | **Diabetes duration, years (T/C)** | **DPN duration, years (T/C)** | **Sex, M/F (T/C)** |
| --- | --- | --- | --- | --- | --- | --- | --- | --- |
| Shen 2009 | TMN | 50 / 50 | NR | NR | 60.0±4.2 / 61.0±5.3 | 8.5 / 7.9 | NR | 21/29 / 27/23 |
| Gao 2012 | RGXFTL | 30 / 30 | NR | NR | NR | NR | NR | 16/14 / 17/13 |
| Zhou 2014 | XMZT | 52 / 50 | NR | NR | 56.4±10.1 / 54.9±9.8 | 7.6±3.9 / 7.3±3.7 | NR | 29/23 / 28/22 |
| Song 2015 | JWGSZM | 30 / 30 | NR | NR | 59.1±5.9 / 59.2±9.9 | NR | 54.5±37.3 months / 54.3±36.7 months | 15/15 / 13/17 |
| Liu 2015 | JWHQGZ | 45 / 45 | NR | NR | 67.98±6.54 / 67.98±6.54 | 8.76±5.13 / 8.76±5.13 | NR | 47/43 / 47/43 |
| Wang 2017 | DGSN | 30 / 30 | 6.32±1.15 / 6.34±1.02 | NR | 58.76±4.32 / 57.21±3.56 | 12.01±2.31 / 11.95±3.14 | 3.56±1.21 / 3.84±1.36 | 15/15 / 16/14 |
| Fang 2017 | YQHX | 31 / 32 | 7.9±1.68 / 7.7±1.85 | NR | 69.57±9.30 / 67.93±8.24 | 11.93±7.82 / 12.76±8.26 | NR | NR |
| Zhang 2018 | JTT | 43 / 44 | NR | NR | 55.16±3.23 / 54.37±4.28 | 11.12±4.53 / 11.39±4.43 | 3.43±1.27 / 3.14±1.51 | 20/23 / 22/22 |
| Pei 2019 | JWBYHW | 34 / 34 | NR | NR | 61.20±8.33 / 60.32±7.96 | 11.23±4.41 / 11.68±4.33 | 2.11±1.65 / 2.20±1.58 | 16/18 / 14/20 |
| Zhao 2019 | DGSN | 50 / 50 | NR | NR | 64.58±7.95 / 65.12±8.21 | 12.21±6.44 / 12.54±6.41 | NR | 30/20 / 28/22 |
| Zhi 2020 | HLZX | 40 / 40 | 7.22±0.38 / 7.54±0.33 | 7.57±0.32 / 7.42±0.31 | 61.25±4.34 / 62.46±4.53 | 7.45±2.15 / 8.35±3.24 | NR | 22/18 / 18/22 |
| Bao 2020 | YQTLHX | 48 / 48 | NR | NR | 61.23±3.62 / 61.53±3.64 | NR | NR | 25/23 / 26/22 |
| Huang 2021 | BQTL | 50 / 50 | NR | NR | 59.83±6.58 / 59.32±7.52 | NR | NR | 23/27 / 21/29 |
| Zhu 2023 | SYSH | 73 / 73 | NR | NR | 53.81±4.46 / 54.08±4.78 | 9.65±2.24 / 9.72±2.18 | 3.34±0.98 / 3.41±0.82 | 31/42 / 34/39 |
| Lei 2024 | YQHSTB | 30 / 30 | NR | NR | 72.3±8.2 / 72.3±8.8 | NR | 13.4±5.6 / 14.2±4.9 | 14/16 / 15/15 |
| Jia 2025 | HQGZWW | 54 / 54 | NR | NR | 50.07±8.14 / 50.18±8.23 | NR | NR | 33/22 / 35/19 |
| Deng 2025 | JWBYHW | 62 / 62 | 8.97±5.27 / 8.83±1.62 | 10.98±1.25 / 10.95±1.18 | 49.81±5.61 / 49.17±4.92 | NR | NR | 35/27 / 37/25 |
| Zhao 2025 | TBF | 93 / 93 | Median 7.60 / Median 7.60 | Median 7.34 / Median 8.28 | 57.4±8.54 / 57.6±7.69 | 10.9±6.72 / 11.2±6.00 | 2.45±2.81 / 2.30±2.35 | 55/38 / 60/33 |
| Jin 2026 | MDKL | 183 / 174 | NR | NR | 58.51±7.97 / 58.41±8.26 | Median 12.0 / Median 10.0 | Median 2.0 / Median 2.0 | 108/75 / 110/64 |

## Table S3b. Baseline severity measures of the included trials

| **Study** | **Node** | **Formula** | **Baseline TCSS, points (T/C)** | **Baseline median MCV, m/s (T/C)** | **Baseline median SCV, m/s (T/C)** | **Baseline peroneal MCV, m/s (T/C)** | **Baseline peroneal SCV, m/s (T/C)** |
| --- | --- | --- | --- | --- | --- | --- | --- |
| Shen 2009 | TMN | Tangmaining Capsule | NR | 41.2±2.6 / 41.8±2.7 | 35.8±2.7 / 36.1±2.6 | 37.2±2.3 / 36.8±2.7 | 32.2±2.9 / 31.6±2.3 |
| Gao 2012 | RGXFTL | Rougan Xifeng Tongluo Decoction | NR | 41.9±1.8 / 41.7±2.0 | 41.6±2.2 / 41.4±2.2 | 37.6±2.1 / 37.0±2.0 | 39.4±2.1 / 40.4±1.6 |
| Zhou 2014 | XMZT | Xiaoma Zhitong Capsule | 9.55±1.69 / 9.59±1.73 | NR | 39.6±4.2 / 39.3±4.1 | 38.6±4.1 / 38.2±4.2 | 33.6±3.8 / 33.4±3.6 |
| Song 2015 | JWGSZM | Modified Guizhi Shaoyao Zhimu Decoction | 10.5±2.4 / 10.6±2.5 | NR | 44.2±6.9 / 44.4±7.6 | 37.4±3.7 / 37.0±3.8 | NR |
| Liu 2015 | JWHQGZ | Modified Huangqi Guizhi Wuwu Decoction | 10.37±2.68 / 10.39±2.71 | 40.67±5.46 / 40.84±5.13 | NR | 35.96±4.18 / 36.32±4.21 | NR |
| Wang 2017 | DGSN | Danggui Sini Decoction | NR | 40.23±4.12 / 39.62±4.92 | 41.87±3.25 / 40.72±2.88 | 35.65±6.71 / 35.81±5.82 | NR |
| Fang 2017 | YQHX | Yiqi Huoxue Formula | 12.49±3.26 / 12.84±2.95 | 35.16±3.27 / 34.45±4.03 | 34.52±2.37 / 36.21±3.24 | NR | NR |
| Zhang 2018 | JTT | Jiangtang Tongluo Tablet | NR | 39.78±4.20 / 37.70±4.16 | 36.49±4.12 / 37.35±4.24 | 38.62±4.15 / 37.56±4.10 | 41.20±3.65 / 40.65±3.54 |
| Pei 2019 | JWBYHW | Modified Buyang Huanwu Decoction | 11.18±2.65 / 11.24±2.73 | 41.97±5.53 / 42.41±6.08 | NR | NR | NR |
| Zhao 2019 | DGSN | Danggui Sini Decoction | 13.16±2.33 / 13.11±1.77 | NR | NR | NR | NR |
| Zhi 2020 | HLZX | Huoluo Zhixiao Formula | NR | 39.2±2.8 / 37.8±2.5 | 35.7±1.4 / 36.0±1.4 | 36.9±2.3 / 35.9±2.3 | 31.1±1.6 / 30.3±1.2 |
| Bao 2020 | YQTLHX | Yiqi Tongluo Huoxue Decoction | 10.36±2.45 / 10.44±2.51 | 39.01±3.18 / 38.76±3.54 | 30.53±2.54 / 30.44±2.61 | 40.36±6.56 / 39.96±6.43 | 41.29±4.17 / 40.34±4.11 |
| Huang 2021 | BQTL | Buqi Tongluo Capsule | 10.35±1.29 / 10.42±1.56 | 38.42±1.87 / 38.56±2.03 | 41.65±2.31 / 41.82±2.24 | NR | 42.19±2.48 / 42.41±2.10 |
| Zhu 2023 | SYSH | Shengyang Sanhuo Decoction | NR | 38.86±2.33 / 38.89±2.27 | 38.32±2.41 / 38.29±2.27 | 38.12±3.37 / 38.08±3.46 | 39.26±2.47 / 39.17±2.35 |
| Lei 2024 | YQHSTB | Yiqi Huashi Tongbi Formula | 11.6±1.6 / 11.3±1.1 | NR | 42.9±3.5 / 43.9±2.9 | 37.3±2.3 / 37.5±1.8 | NR |
| Jia 2025 | HQGZWW | Huangqi Guizhi Wuwu Decoction | 9.58±0.91 / 9.68±0.95 | 40.65±4.18 / 40.46±3.83 | NR | NR | 34.53±2.91 / 34.41±2.82 |
| Deng 2025 | JWBYHW | Modified Buyang Huanwu Decoction | NR | 39.97±3.49 / 39.43±3.60 | NR | 38.23±4.60 / 37.91±4.87 | NR |
| Zhao 2025 | TBF | TangBi Formula | 7.7±2.76 / 7.3±2.48 | 52.21±8.17 / 53.75±5.68 | 48.25±8.89 / 49.15±7.97 | 43.10±7.88 / 42.79±7.30 | NR |
| Jin 2026 | MDKL | Mudan Granule | 9.08±2.93 / 8.99±2.77 | 53.32±5.87 / 53.09±6.74 | 47.07±9.18 / 47.11±9.36 | 44.75±6.13 / 44.63±5.65 | NR |

## Table S3c. Baseline severity by treatment node

| **Outcome** | **Unit** | **Node** | **Formula** | **Contributing trial(s)** | **Baseline value, mean** |
| --- | --- | --- | --- | --- | --- |
| TCSS total score | points | YQHSTB | Yiqi Huashi Tongbi Formula | Lei 2024 | 11.45 |
| TCSS total score | points | HQGZWW | Huangqi Guizhi Wuwu Decoction | Jia 2025 | 9.63 |
| TCSS total score | points | JWBYHW | Modified Buyang Huanwu Decoction | Pei 2019 | 11.21 |
| TCSS total score | points | MDKL | Mudan Granule | Jin 2026 | 9.04 |
| TCSS total score | points | JWHQGZ | Modified Huangqi Guizhi Wuwu Decoction | Liu 2015 | 10.38 |
| TCSS total score | points | BQTL | Buqi Tongluo Capsule | Huang 2021 | 10.38 |
| TCSS total score | points | YQTLHX | Yiqi Tongluo Huoxue Decoction | Bao 2020 | 10.40 |
| TCSS total score | points | DGSN | Danggui Sini Decoction | Zhao 2019 | 13.13 |
| TCSS total score | points | JWGSZM | Modified Guizhi Shaoyao Zhimu Decoction | Song 2015 | 10.55 |
| TCSS total score | points | XMZT | Xiaoma Zhitong Capsule | Zhou 2014 | 9.57 |
| TCSS total score | points | YQHX | Yiqi Huoxue Formula | Fang 2017 | 12.67 |
| TCSS total score | points | TBF | TangBi Formula | Zhao 2025 | 7.50 |
| Median nerve MCV | m/s | SYSH | Shengyang Sanhuo Decoction | Zhu 2023 | 38.88 |
| Median nerve MCV | m/s | YQHX | Yiqi Huoxue Formula | Fang 2017 | 34.80 |
| Median nerve MCV | m/s | JWHQGZ | Modified Huangqi Guizhi Wuwu Decoction | Liu 2015 | 40.76 |
| Median nerve MCV | m/s | BQTL | Buqi Tongluo Capsule | Huang 2021 | 38.49 |
| Median nerve MCV | m/s | HLZX | Huoluo Zhixiao Formula | Zhi 2020 | 38.50 |
| Median nerve MCV | m/s | HQGZWW | Huangqi Guizhi Wuwu Decoction | Jia 2025 | 40.56 |
| Median nerve MCV | m/s | YQTLHX | Yiqi Tongluo Huoxue Decoction | Bao 2020 | 38.88 |
| Median nerve MCV | m/s | JWBYHW | Modified Buyang Huanwu Decoction | Deng 2025 + Pei 2019 | 40.58 |
| Median nerve MCV | m/s | TMN | Tangmaining Capsule | Shen 2009 | 41.50 |
| Median nerve MCV | m/s | JTT | Jiangtang Tongluo Tablet | Zhang 2018 | 38.73 |
| Median nerve MCV | m/s | RGXFTL | Rougan Xifeng Tongluo Decoction | Gao 2012 | 41.80 |
| Median nerve MCV | m/s | TBF | TangBi Formula | Zhao 2025 | 52.98 |
| Median nerve MCV | m/s | DGSN | Danggui Sini Decoction | Wang 2017 | 39.92 |
| Median nerve MCV | m/s | MDKL | Mudan Granule | Jin 2026 | 53.21 |
| Median nerve SCV | m/s | XMZT | Xiaoma Zhitong Capsule | Zhou 2014 | 39.45 |
| Median nerve SCV | m/s | JTT | Jiangtang Tongluo Tablet | Zhang 2018 | 36.92 |
| Median nerve SCV | m/s | YQHSTB | Yiqi Huashi Tongbi Formula | Lei 2024 | 43.40 |
| Median nerve SCV | m/s | BQTL | Buqi Tongluo Capsule | Huang 2021 | 41.73 |
| Median nerve SCV | m/s | SYSH | Shengyang Sanhuo Decoction | Zhu 2023 | 38.31 |
| Median nerve SCV | m/s | YQHX | Yiqi Huoxue Formula | Fang 2017 | 35.38 |
| Median nerve SCV | m/s | RGXFTL | Rougan Xifeng Tongluo Decoction | Gao 2012 | 41.50 |
| Median nerve SCV | m/s | TMN | Tangmaining Capsule | Shen 2009 | 35.95 |
| Median nerve SCV | m/s | JWGSZM | Modified Guizhi Shaoyao Zhimu Decoction | Song 2015 | 44.30 |
| Median nerve SCV | m/s | YQTLHX | Yiqi Tongluo Huoxue Decoction | Bao 2020 | 30.49 |
| Median nerve SCV | m/s | HLZX | Huoluo Zhixiao Formula | Zhi 2020 | 35.85 |
| Median nerve SCV | m/s | MDKL | Mudan Granule | Jin 2026 | 47.09 |
| Median nerve SCV | m/s | TBF | TangBi Formula | Zhao 2025 | 48.70 |
| Median nerve SCV | m/s | DGSN | Danggui Sini Decoction | Wang 2017 | 41.29 |
| Peroneal nerve MCV | m/s | JWBYHW | Modified Buyang Huanwu Decoction | Deng 2025 | 38.07 |
| Peroneal nerve MCV | m/s | JWHQGZ | Modified Huangqi Guizhi Wuwu Decoction | Liu 2015 | 36.14 |
| Peroneal nerve MCV | m/s | DGSN | Danggui Sini Decoction | Wang 2017 | 35.73 |
| Peroneal nerve MCV | m/s | JWGSZM | Modified Guizhi Shaoyao Zhimu Decoction | Song 2015 | 37.20 |
| Peroneal nerve MCV | m/s | YQHSTB | Yiqi Huashi Tongbi Formula | Lei 2024 | 37.40 |
| Peroneal nerve MCV | m/s | JTT | Jiangtang Tongluo Tablet | Zhang 2018 | 38.08 |
| Peroneal nerve MCV | m/s | SYSH | Shengyang Sanhuo Decoction | Zhu 2023 | 38.10 |
| Peroneal nerve MCV | m/s | YQTLHX | Yiqi Tongluo Huoxue Decoction | Bao 2020 | 40.16 |
| Peroneal nerve MCV | m/s | HLZX | Huoluo Zhixiao Formula | Zhi 2020 | 36.40 |
| Peroneal nerve MCV | m/s | RGXFTL | Rougan Xifeng Tongluo Decoction | Gao 2012 | 37.30 |
| Peroneal nerve MCV | m/s | XMZT | Xiaoma Zhitong Capsule | Zhou 2014 | 38.40 |
| Peroneal nerve MCV | m/s | TMN | Tangmaining Capsule | Shen 2009 | 37.00 |
| Peroneal nerve MCV | m/s | TBF | TangBi Formula | Zhao 2025 | 42.95 |
| Peroneal nerve MCV | m/s | MDKL | Mudan Granule | Jin 2026 | 44.69 |
| Peroneal nerve SCV | m/s | JTT | Jiangtang Tongluo Tablet | Zhang 2018 | 40.92 |
| Peroneal nerve SCV | m/s | SYSH | Shengyang Sanhuo Decoction | Zhu 2023 | 39.22 |
| Peroneal nerve SCV | m/s | BQTL | Buqi Tongluo Capsule | Huang 2021 | 42.30 |
| Peroneal nerve SCV | m/s | HQGZWW | Huangqi Guizhi Wuwu Decoction | Jia 2025 | 34.47 |
| Peroneal nerve SCV | m/s | YQTLHX | Yiqi Tongluo Huoxue Decoction | Bao 2020 | 40.82 |
| Peroneal nerve SCV | m/s | HLZX | Huoluo Zhixiao Formula | Zhi 2020 | 30.70 |
| Peroneal nerve SCV | m/s | TMN | Tangmaining Capsule | Shen 2009 | 31.90 |
| Peroneal nerve SCV | m/s | RGXFTL | Rougan Xifeng Tongluo Decoction | Gao 2012 | 39.90 |
| Peroneal nerve SCV | m/s | XMZT | Xiaoma Zhitong Capsule | Zhou 2014 | 33.50 |

## Table S3d. Distribution of potential effect modifiers

| **Potential effect modifier** | **Unit** | **Analysis set** | **Trials analysed** | **Trials without data** | **Trials without data, %** | **Minimum** | **Maximum** | **Median** | **Range** | **Coefficient of variation** |
| --- | --- | --- | --- | --- | --- | --- | --- | --- | --- | --- |
| Baseline TCSS total score | points | Trials contributing to the TCSS total score network | 12 | 0 | 0% | 7.50 | 13.13 | 10.39 | 5.63 | 14.6% |
| Baseline Median nerve MCV | m/s | Trials contributing to the Median nerve MCV network | 15 | 0 | 0% | 34.80 | 53.21 | 39.92 | 18.41 | 12.3% |
| Baseline Median nerve SCV | m/s | Trials contributing to the Median nerve SCV network | 14 | 0 | 0% | 30.49 | 48.70 | 40.37 | 18.21 | 12.4% |
| Baseline Peroneal nerve MCV | m/s | Trials contributing to the Peroneal nerve MCV network | 14 | 0 | 0% | 35.73 | 44.69 | 37.73 | 8.96 | 6.7% |
| Baseline Peroneal nerve SCV | m/s | Trials contributing to the Peroneal nerve SCV network | 9 | 0 | 0% | 30.70 | 42.30 | 39.22 | 11.60 | 11.9% |
| Baseline HbA1c | % | All 19 included trials | 4 | 15 | 79% | 6.33 | 8.90 | 7.59 | 2.57 | 14.0% |
| Baseline FPG | mmol/L | All 19 included trials | 2 | 17 | 89% | 7.50 | 10.96 | 9.23 | 3.47 | 26.6% |
| Age | years | All 19 included trials | 18 | 1 | 5% | 49.49 | 72.30 | 59.36 | 22.81 | 10.1% |
| Diabetes duration | years | All 19 included trials | 11 | 8 | 42% | 7.45 | 12.38 | 11.05 | 4.92 | 18.3% |
| DPN duration | years | All 19 included trials | 7 | 12 | 63% | 2.16 | 13.80 | 3.38 | 11.64 | 85.8% |

## Table S4. Funnel plot degeneracy

| **Outcome** | **Trials** | **Non-zero residuals** | **Largest absolute residual** |
| --- | --- | --- | --- |
| TCSS total score | 12 | 0 | 0 |
| Median nerve MCV | 15 | 2 | 1.2448 |
| Median nerve SCV | 14 | 0 | 0 |
| Peroneal nerve MCV | 14 | 0 | 0 |
| Peroneal nerve SCV | 9 | 0 | 0 |

## Table S5. Pairwise meta-analyses

| **Outcome** | **Trials (k)** | **MD (random effects)** | **95% CI** | **Method used for the confidence interval** | **Test statistic for the pooled effect (t)** | **Degrees of freedom for the pooled effect** | **P value for the pooled effect** | **Cochran Q** | **Degrees of freedom for heterogeneity** | **P value for heterogeneity** | **tau squared** | **I2 (%)** | **Prediction interval** |
| --- | --- | --- | --- | --- | --- | --- | --- | --- | --- | --- | --- | --- | --- |
| TCSS total score | 12 | -2.136 | -2.662 to -1.61 | Knapp-Hartung t | -8.938 | 11 | <0.001 | 115.99 | 11 | <0.001 | 0.5589 | 90.5 | -3.864 to -0.409 |
| Median nerve MCV | 15 | 2.389 | 1.664 to 3.114 | Knapp-Hartung t | 7.066 | 14 | <0.001 | 80.639 | 14 | <0.001 | 1.3061 | 82.6 | -0.167 to 4.944 |
| Median nerve SCV | 14 | 2.056 | 1.135 to 2.977 | Knapp-Hartung t | 4.821 | 13 | <0.001 | 93.102 | 13 | <0.001 | 2.1008 | 86 | -1.208 to 5.32 |
| Peroneal nerve MCV | 14 | 2.132 | 1.284 to 2.98 | Knapp-Hartung t | 5.429 | 13 | <0.001 | 76.173 | 13 | <0.001 | 1.6364 | 82.9 | -0.759 to 5.023 |
| Peroneal nerve SCV | 9 | 2.325 | 1.71 to 2.94 | Knapp-Hartung t | 8.715 | 8 | <0.001 | 24.074 | 8 | 0.0022 | 0.3781 | 66.8 | 0.79 to 3.86 |

## Table S6a. Posterior between-study standard deviation under alternative priors

| **Outcome** | **Model** | **tau_post_mean** | **tau_lo** | **tau_hi** | **tau_pairwise_REML** |
| --- | --- | --- | --- | --- | --- |
| TCSS total score | B. Random effects, half-normal prior, SD = 0.748 | 0.5951 | 0.0235 | 1.6695 | 0.7476 |
| TCSS total score | C. Random effects, uniform prior U(0, 2.24) | 1.1163 | 0.0512 | 2.1854 | 0.7476 |
| Median nerve MCV | B. Random effects, half-normal prior, SD = 1.143 | 0.9886 | 0.049 | 2.5397 | 1.1429 |
| Median nerve MCV | C. Random effects, uniform prior U(0, 3.43) | 1.6872 | 0.1075 | 3.32 | 1.1429 |
| Median nerve SCV | B. Random effects, half-normal prior, SD = 1.449 | 1.1622 | 0.0537 | 3.2446 | 1.4494 |
| Median nerve SCV | C. Random effects, uniform prior U(0, 4.35) | 2.147 | 0.0988 | 4.2349 | 1.4494 |
| Peroneal nerve MCV | B. Random effects, half-normal prior, SD = 1.279 | 1.0194 | 0.0396 | 2.8252 | 1.2792 |
| Peroneal nerve MCV | C. Random effects, uniform prior U(0, 3.84) | 1.8874 | 0.0805 | 3.7374 | 1.2792 |
| Peroneal nerve SCV | B. Random effects, half-normal prior, SD = 0.615 | 0.4913 | 0.0191 | 1.3756 | 0.6149 |
| Peroneal nerve SCV | C. Random effects, uniform prior U(0, 2.00) | 0.9966 | 0.0484 | 1.9507 | 0.6149 |

## Table S6b. Identifiability of the between-study standard deviation

| **Outcome** | **Trials** | **Treatment nodes** | **Degrees of freedom for heterogeneity (df.Q)** | **Network tau estimable** | **Network tau** | **Network I2 (%)** | **Pairwise tau (REML)** | **Pairwise I2 (%)** |
| --- | --- | --- | --- | --- | --- | --- | --- | --- |
| TCSS total score | 12 | 13 | 0 | No (no repeated comparison) | Not estimable | Not estimable | 0.7476 | 90.5 |
| Median nerve MCV | 15 | 15 | 1 | Yes | 1.2851 | 70.8 | 1.1429 | 82.6 |
| Median nerve SCV | 14 | 15 | 0 | No (no repeated comparison) | Not estimable | Not estimable | 1.4494 | 86 |
| Peroneal nerve MCV | 14 | 15 | 0 | No (no repeated comparison) | Not estimable | Not estimable | 1.2792 | 82.9 |
| Peroneal nerve SCV | 9 | 10 | 0 | No (no repeated comparison) | Not estimable | Not estimable | 0.6149 | 66.8 |

## Table S7a. Convergence diagnostics

| **Outcome** | **Model** | **Maximum PSRF** | **Minimum effective sample size** | **Maximum Monte Carlo standard error** | **Maximum MCSE relative to the posterior SD** | **Dbar** | **pD** | **DIC** |
| --- | --- | --- | --- | --- | --- | --- | --- | --- |
| TCSS total score | A. Common effect | 1.0003 | 38109 | 0.00284 | 0.0051 | 24.13 | 24.13 | 48.26 |
| TCSS total score | B. Random effects, half-normal prior, SD = 0.748 | 1.0005 | 7815 | 0.00522 | 0.0113 | 24.01 | 24.01 | 48.03 |
| TCSS total score | C. Random effects, uniform prior U(0, 2.24) | 1.0005 | 6978 | 0.00785 | 0.0121 | 24.05 | 24.05 | 48.1 |
| Median nerve MCV | A. Common effect | 1.0002 | 38581 | 0.00436 | 0.0051 | 32.51 | 29.08 | 61.59 |
| Median nerve MCV | B. Random effects, half-normal prior, SD = 1.143 | 1.0005 | 7336 | 0.00832 | 0.0117 | 30.62 | 29.74 | 60.36 |
| Median nerve MCV | C. Random effects, uniform prior U(0, 3.43) | 1.0006 | 7007 | 0.0111 | 0.012 | 30.28 | 29.95 | 60.23 |
| Median nerve SCV | A. Common effect | 1.0004 | 38829 | 0.00818 | 0.0051 | 28.02 | 28.02 | 56.04 |
| Median nerve SCV | B. Random effects, half-normal prior, SD = 1.449 | 1.0004 | 6649 | 0.01504 | 0.0123 | 28.03 | 28.03 | 56.07 |
| Median nerve SCV | C. Random effects, uniform prior U(0, 4.35) | 1.0003 | 6853 | 0.01601 | 0.0121 | 28.04 | 28.04 | 56.08 |
| Peroneal nerve MCV | A. Common effect | 1.0003 | 38213 | 0.00788 | 0.0051 | 27.97 | 27.97 | 55.93 |
| Peroneal nerve MCV | B. Random effects, half-normal prior, SD = 1.279 | 1.0009 | 6863 | 0.0142 | 0.0121 | 28.01 | 28 | 56.01 |
| Peroneal nerve MCV | C. Random effects, uniform prior U(0, 3.84) | 1.001 | 6132 | 0.01489 | 0.0128 | 27.97 | 27.97 | 55.94 |
| Peroneal nerve SCV | A. Common effect | 1.0002 | 39251 | 0.00389 | 0.005 | 18.02 | 18.02 | 36.04 |
| Peroneal nerve SCV | B. Random effects, half-normal prior, SD = 0.615 | 1.0013 | 9879 | 0.00672 | 0.0101 | 17.99 | 17.99 | 35.98 |
| Peroneal nerve SCV | C. Random effects, uniform prior U(0, 2.00) | 1.0013 | 9455 | 0.00733 | 0.0103 | 18.05 | 18.05 | 36.1 |

## Table S7b. Residual deviance

| **Outcome** | **Model** | **Data points** | **Total residual deviance** | **Mean residual deviance per data point** |
| --- | --- | --- | --- | --- |
| TCSS total score | A. Common effect | 24 | 24.13 | 1.006 |
| TCSS total score | B. Random effects, half-normal prior, SD = 0.748 | 24 | 24.01 | 1.001 |
| TCSS total score | C. Random effects, uniform prior U(0, 2.24) | 24 | 24.05 | 1.002 |
| Median nerve MCV | A. Common effect | 30 | 32.51 | 1.084 |
| Median nerve MCV | B. Random effects, half-normal prior, SD = 1.143 | 30 | 30.62 | 1.021 |
| Median nerve MCV | C. Random effects, uniform prior U(0, 3.43) | 30 | 30.28 | 1.009 |
| Median nerve SCV | A. Common effect | 28 | 28.02 | 1.001 |
| Median nerve SCV | B. Random effects, half-normal prior, SD = 1.449 | 28 | 28.03 | 1.001 |
| Median nerve SCV | C. Random effects, uniform prior U(0, 4.35) | 28 | 28.04 | 1.001 |
| Peroneal nerve MCV | A. Common effect | 28 | 27.97 | 0.999 |
| Peroneal nerve MCV | B. Random effects, half-normal prior, SD = 1.279 | 28 | 28.01 | 1 |
| Peroneal nerve MCV | C. Random effects, uniform prior U(0, 3.84) | 28 | 27.97 | 0.999 |
| Peroneal nerve SCV | A. Common effect | 18 | 18.02 | 1.001 |
| Peroneal nerve SCV | B. Random effects, half-normal prior, SD = 0.615 | 18 | 17.99 | 0.999 |
| Peroneal nerve SCV | C. Random effects, uniform prior U(0, 2.00) | 18 | 18.05 | 1.003 |

## Table S8. Sensitivity analysis: assumed pre-post correlation

| **Outcome** | **Assumed pre-post correlation r** | **Top-ranked formula** | **MD, frequentist network model** | **95% CI, frequentist network model** | **Width of the 95% CI** |
| --- | --- | --- | --- | --- | --- |
| TCSS total score | 0.5 | YQHSTB | -3.5 | -4.242 to -2.758 | 1.484 |
| TCSS total score | 0.6 | YQHSTB | -3.5 | -4.164 to -2.836 | 1.327 |
| TCSS total score | 0.7 | YQHSTB | -3.5 | -4.075 to -2.925 | 1.149 |
| TCSS total score | 0.8 | YQHSTB | -3.5 | -3.969 to -3.031 | 0.938 |
| TCSS total score | 0.9 | YQHSTB | -3.5 | -3.832 to -3.168 | 0.664 |
| Median nerve MCV | 0.5 | SYSH | 4.16 | 1.897 to 6.423 | 4.526 |
| Median nerve MCV | 0.6 | SYSH | 4.16 | 1.733 to 6.587 | 4.854 |
| Median nerve MCV | 0.7 | SYSH | 4.16 | 1.580 to 6.740 | 5.16 |
| Median nerve MCV | 0.8 | SYSH | 4.16 | 1.435 to 6.885 | 5.45 |
| Median nerve MCV | 0.9 | SYSH | 4.16 | 1.298 to 7.022 | 5.725 |
| Median nerve SCV | 0.5 | XMZT | 4.2 | 2.418 to 5.982 | 3.564 |
| Median nerve SCV | 0.6 | XMZT | 4.2 | 2.606 to 5.794 | 3.187 |
| Median nerve SCV | 0.7 | XMZT | 4.2 | 2.820 to 5.580 | 2.76 |
| Median nerve SCV | 0.8 | XMZT | 4.2 | 3.073 to 5.327 | 2.254 |
| Median nerve SCV | 0.9 | XMZT | 4.2 | 3.403 to 4.997 | 1.594 |
| Peroneal nerve MCV | 0.5 | JWBYHW | 3.78 | 2.237 to 5.323 | 3.087 |
| Peroneal nerve MCV | 0.6 | JWBYHW | 3.78 | 2.399 to 5.161 | 2.761 |
| Peroneal nerve MCV | 0.7 | JWBYHW | 3.78 | 2.584 to 4.976 | 2.391 |
| Peroneal nerve MCV | 0.8 | JWBYHW | 3.78 | 2.804 to 4.756 | 1.952 |
| Peroneal nerve MCV | 0.9 | JWBYHW | 3.78 | 3.090 to 4.470 | 1.381 |
| Peroneal nerve SCV | 0.5 | JTT | 4.09 | 2.587 to 5.593 | 3.006 |
| Peroneal nerve SCV | 0.6 | JTT | 4.09 | 2.746 to 5.434 | 2.689 |
| Peroneal nerve SCV | 0.7 | JTT | 4.09 | 2.926 to 5.254 | 2.328 |
| Peroneal nerve SCV | 0.8 | JTT | 4.09 | 3.139 to 5.041 | 1.901 |
| Peroneal nerve SCV | 0.9 | JTT | 4.09 | 3.418 to 4.762 | 1.344 |

## Table S9a. Leave-one-out analysis

| **Outcome** | **Trial omitted** | **Node omitted** | **Top-ranked in the full network** | **Top-ranked after omission** | **Top-ranked treatment changed** | **Largest change in MD among the remaining nodes** | **Network tau after omission** |
| --- | --- | --- | --- | --- | --- | --- | --- |
| TCSS total score | Lei 2024 | YQHSTB | YQHSTB | HQGZWW | Yes | 0 | Not estimable |
| TCSS total score | Jia 2025 | HQGZWW | YQHSTB | YQHSTB | No | 0 | Not estimable |
| TCSS total score | Pei 2019 | JWBYHW | YQHSTB | YQHSTB | No | 0 | Not estimable |
| TCSS total score | Liu 2015 | JWHQGZ | YQHSTB | YQHSTB | No | 0 | Not estimable |
| TCSS total score | Jin 2026 | MDKL | YQHSTB | YQHSTB | No | 0 | Not estimable |
| TCSS total score | Huang 2021 | BQTL | YQHSTB | YQHSTB | No | 0 | Not estimable |
| TCSS total score | Bao 2020 | YQTLHX | YQHSTB | YQHSTB | No | 0 | Not estimable |
| TCSS total score | Zhao 2019 | DGSN | YQHSTB | YQHSTB | No | 0 | Not estimable |
| TCSS total score | Zhou 2014 | XMZT | YQHSTB | YQHSTB | No | 0 | Not estimable |
| TCSS total score | Song 2015 | JWGSZM | YQHSTB | YQHSTB | No | 0 | Not estimable |
| TCSS total score | Fang 2017 | YQHX | YQHSTB | YQHSTB | No | 0 | Not estimable |
| TCSS total score | Zhao 2025 | TBF | YQHSTB | YQHSTB | No | 0 | Not estimable |
| Median nerve MCV | Jin 2026 | MDKL | SYSH | SYSH | No | 0 | 1.2851 |
| Median nerve MCV | Wang 2017 | DGSN | SYSH | SYSH | No | 0 | 1.2851 |
| Median nerve MCV | Zhao 2025 | TBF | SYSH | SYSH | No | 0 | 1.2851 |
| Median nerve MCV | Gao 2012 | RGXFTL | SYSH | SYSH | No | 0 | 1.2851 |
| Median nerve MCV | Deng 2025 | JWBYHW | SYSH | SYSH | No | 1.2448 | Not estimable |
| Median nerve MCV | Zhang 2018 | JTT | SYSH | SYSH | No | 0 | 1.2851 |
| Median nerve MCV | Shen 2009 | TMN | SYSH | SYSH | No | 0 | 1.2851 |
| Median nerve MCV | Bao 2020 | YQTLHX | SYSH | SYSH | No | 0 | 1.2851 |
| Median nerve MCV | Jia 2025 | HQGZWW | SYSH | SYSH | No | 0 | 1.2851 |
| Median nerve MCV | Zhi 2020 | HLZX | SYSH | SYSH | No | 0 | 1.2851 |
| Median nerve MCV | Huang 2021 | BQTL | SYSH | SYSH | No | 0 | 1.2851 |
| Median nerve MCV | Liu 2015 | JWHQGZ | SYSH | SYSH | No | 0 | 1.2851 |
| Median nerve MCV | Pei 2019 | JWBYHW | SYSH | SYSH | No | 0.9152 | Not estimable |
| Median nerve MCV | Fang 2017 | YQHX | SYSH | SYSH | No | 0 | 1.2851 |
| Median nerve MCV | Zhu 2023 | SYSH | SYSH | YQHX | Yes | 0 | 1.2851 |
| Median nerve SCV | Wang 2017 | DGSN | XMZT | XMZT | No | 0 | Not estimable |
| Median nerve SCV | Zhao 2025 | TBF | XMZT | XMZT | No | 0 | Not estimable |
| Median nerve SCV | Jin 2026 | MDKL | XMZT | XMZT | No | 0 | Not estimable |
| Median nerve SCV | Zhi 2020 | HLZX | XMZT | XMZT | No | 0 | Not estimable |
| Median nerve SCV | Song 2015 | JWGSZM | XMZT | XMZT | No | 0 | Not estimable |
| Median nerve SCV | Bao 2020 | YQTLHX | XMZT | XMZT | No | 0 | Not estimable |
| Median nerve SCV | Shen 2009 | TMN | XMZT | XMZT | No | 0 | Not estimable |
| Median nerve SCV | Gao 2012 | RGXFTL | XMZT | XMZT | No | 0 | Not estimable |
| Median nerve SCV | Fang 2017 | YQHX | XMZT | XMZT | No | 0 | Not estimable |
| Median nerve SCV | Zhu 2023 | SYSH | XMZT | XMZT | No | 0 | Not estimable |
| Median nerve SCV | Huang 2021 | BQTL | XMZT | XMZT | No | 0 | Not estimable |
| Median nerve SCV | Lei 2024 | YQHSTB | XMZT | XMZT | No | 0 | Not estimable |
| Median nerve SCV | Zhang 2018 | JTT | XMZT | XMZT | No | 0 | Not estimable |
| Median nerve SCV | Zhou 2014 | XMZT | XMZT | JTT | Yes | 0 | Not estimable |
| Peroneal nerve MCV | Jin 2026 | MDKL | JWBYHW | JWBYHW | No | 0 | Not estimable |
| Peroneal nerve MCV | Zhao 2025 | TBF | JWBYHW | JWBYHW | No | 0 | Not estimable |
| Peroneal nerve MCV | Shen 2009 | TMN | JWBYHW | JWBYHW | No | 0 | Not estimable |
| Peroneal nerve MCV | Zhou 2014 | XMZT | JWBYHW | JWBYHW | No | 0 | Not estimable |
| Peroneal nerve MCV | Gao 2012 | RGXFTL | JWBYHW | JWBYHW | No | 0 | Not estimable |
| Peroneal nerve MCV | Zhi 2020 | HLZX | JWBYHW | JWBYHW | No | 0 | Not estimable |
| Peroneal nerve MCV | Bao 2020 | YQTLHX | JWBYHW | JWBYHW | No | 0 | Not estimable |
| Peroneal nerve MCV | Zhu 2023 | SYSH | JWBYHW | JWBYHW | No | 0 | Not estimable |
| Peroneal nerve MCV | Zhang 2018 | JTT | JWBYHW | JWBYHW | No | 0 | Not estimable |
| Peroneal nerve MCV | Lei 2024 | YQHSTB | JWBYHW | JWBYHW | No | 0 | Not estimable |
| Peroneal nerve MCV | Song 2015 | JWGSZM | JWBYHW | JWBYHW | No | 0 | Not estimable |
| Peroneal nerve MCV | Liu 2015 | JWHQGZ | JWBYHW | JWBYHW | No | 0 | Not estimable |
| Peroneal nerve MCV | Wang 2017 | DGSN | JWBYHW | JWBYHW | No | 0 | Not estimable |
| Peroneal nerve MCV | Deng 2025 | JWBYHW | JWBYHW | JWHQGZ | Yes | 0 | Not estimable |
| Peroneal nerve SCV | Zhou 2014 | XMZT | JTT | JTT | No | 0 | Not estimable |
| Peroneal nerve SCV | Gao 2012 | RGXFTL | JTT | JTT | No | 0 | Not estimable |
| Peroneal nerve SCV | Shen 2009 | TMN | JTT | JTT | No | 0 | Not estimable |
| Peroneal nerve SCV | Zhi 2020 | HLZX | JTT | JTT | No | 0 | Not estimable |
| Peroneal nerve SCV | Bao 2020 | YQTLHX | JTT | JTT | No | 0 | Not estimable |
| Peroneal nerve SCV | Jia 2025 | HQGZWW | JTT | JTT | No | 0 | Not estimable |
| Peroneal nerve SCV | Huang 2021 | BQTL | JTT | JTT | No | 0 | Not estimable |
| Peroneal nerve SCV | Zhu 2023 | SYSH | JTT | JTT | No | 0 | Not estimable |
| Peroneal nerve SCV | Zhang 2018 | JTT | JTT | SYSH | Yes | 0 | Not estimable |

## Table S9b. Node redefinition

| **Outcome** | **Merged node** | **Top-ranked in the full network** | **Top-ranked after splitting** | **Top-ranked treatment changed** |
| --- | --- | --- | --- | --- |
| TCSS total score | None |  |  | Not applicable |
| Median nerve MCV | JWBYHW | SYSH | SYSH | No |
| Median nerve SCV | None |  |  | Not applicable |
| Peroneal nerve MCV | None |  |  | Not applicable |
| Peroneal nerve SCV | None |  |  | Not applicable |

## Table S10. Publication bias

| **Outcome** | **Test** | **Trials (k)** | **Test statistic** | **P value** | **Model** |
| --- | --- | --- | --- | --- | --- |
| TCSS total score | Egger linear regression | 12 | t = 1.953 | 0.0794 | random-effects |
| TCSS total score | Begg-Mazumdar rank correlation | 12 | z = 0.411 | 0.6808 | random-effects |
| TCSS total score | Thompson-Sharp | 12 | t = 1.030 | 0.3274 | random-effects |
| Median nerve MCV | Egger linear regression | 15 | t = -2.017 | 0.0649 | random-effects |
| Median nerve MCV | Begg-Mazumdar rank correlation | 15 | z = -0.544 | 0.5862 | random-effects |
| Median nerve MCV | Thompson-Sharp | 15 | t = -0.630 | 0.5398 | random-effects |
| Median nerve SCV | Egger linear regression | 14 | t = -0.708 | 0.4927 | random-effects |
| Median nerve SCV | Begg-Mazumdar rank correlation | 14 | z = -0.274 | 0.7843 | random-effects |
| Median nerve SCV | Thompson-Sharp | 14 | t = -0.365 | 0.7215 | random-effects |
| Peroneal nerve MCV | Egger linear regression | 14 | t = 0.861 | 0.4061 | random-effects |
| Peroneal nerve MCV | Begg-Mazumdar rank correlation | 14 | z = -0.055 | 0.9563 | random-effects |
| Peroneal nerve MCV | Thompson-Sharp | 14 | t = 1.064 | 0.3085 | random-effects |
| Peroneal nerve SCV | Egger linear regression | 9 | t = -0.118 | 0.9090 | random-effects |
| Peroneal nerve SCV | Begg-Mazumdar rank correlation | 9 | z = -0.625 | 0.5316 | random-effects |
| Peroneal nerve SCV | Thompson-Sharp | 9 | t = -0.030 | 0.9771 | random-effects |
